# Pharmacological targeting of the mitochondrial calcium-dependent potassium channel KCa3.1 triggers cell death and reduces tumor growth and metastasis in vivo

**DOI:** 10.1038/s41419-022-05463-8

**Published:** 2022-12-20

**Authors:** Magdalena Bachmann, Andrea Rossa, Tatiana Varanita, Bernard Fioretti, Lucia Biasutto, Stefan Milenkovic, Vanessa Checchetto, Roberta Peruzzo, Syed A. Ahmad, Sameer H. Patel, Robert Lukowski, Michael J. Edwards, Matteo Ceccarelli, Erich Gulbins, Mario Zoratti, Andrea Mattarei, Ildiko Szabo

**Affiliations:** 1 Department of Biology, University of Padova, Padova, Italy; 2 Department of Chemical Sciences, University of Padova, Padova, Italy; 3 Department of Chemistry, Biology and Biotechnology, University of Perugia, Perugia, Italy; 4 CNR Institute of Neuroscience, Padova, Italy; 5 CNR Institute of Materials, Cagliari, Italy; 6 Department of Surgery, University of Cincinnati, Cincinnati, USA; 7 Department of Pharmacology, Toxicology and Clinical Pharmacy, University of Tübingen, Tübingen, Germany; 8 Department of Physics, University of Cagliari, Monserrato, Italy; 9 Department of Molecular Biology, University of Duisburg-Essen, Essen, Germany; 10 Department of Pharmaceutical and Pharmacological Sciences, University of Padova, Padova, Italy

**Keywords:** Cell migration, Cancer therapy

## Abstract

Ion channels are non-conventional, druggable oncological targets. The intermediate-conductance calcium-dependent potassium channel (K_Ca_3.1) is highly expressed in the plasma membrane and in the inner mitochondrial membrane (mitoK_Ca_3.1) of various cancer cell lines. The role mitoK_Ca_3.1 plays in cancer cells is still undefined. Here we report the synthesis and characterization of two mitochondria-targeted novel derivatives of a high-affinity K_Ca_3.1 antagonist, TRAM-34, which retain the ability to block channel activity. The effects of these drugs were tested in melanoma, pancreatic ductal adenocarcinoma and breast cancer lines, as well as in vivo in two orthotopic models. We show that the mitochondria-targeted TRAM-34 derivatives induce release of mitochondrial reactive oxygen species, rapid depolarization of the mitochondrial membrane, fragmentation of the mitochondrial network. They trigger cancer cell death with an EC_50_ in the µM range, depending on channel expression. In contrast, inhibition of the plasma membrane K_Ca_3.1 by membrane-impermeant Maurotoxin is without effect, indicating a specific role of mitoK_Ca_3.1 in determining cell fate. At sub-lethal concentrations, pharmacological targeting of mitoK_Ca_3.1 significantly reduced cancer cell migration by enhancing production of mitochondrial reactive oxygen species and nuclear factor-κB (NF-κB) activation, and by downregulating expression of Bcl-2 Nineteen kD-Interacting Protein (BNIP-3) and of Rho GTPase CDC-42. This signaling cascade finally leads to cytoskeletal reorganization and impaired migration. Overexpression of BNIP-3 or pharmacological modulation of NF-κB and CDC-42 prevented the migration-reducing effect of mitoTRAM-34. In orthotopic models of melanoma and pancreatic ductal adenocarcinoma, the tumors at sacrifice were 60% smaller in treated versus untreated animals. Metastasis of melanoma cells to lymph nodes was also drastically reduced. No signs of toxicity were observed. In summary, our results identify mitochondrial K_Ca_3.1 as an unexpected player in cancer cell migration and show that its pharmacological targeting is efficient against both tumor growth and metastatic spread in vivo.

## Introduction

Mitochondrial ion channels are emerging targets in various pathologies, including cancer and neurodegenerative and cardiovascular diseases [1]. Perturbation of ion fluxes across the outer and inner mitochondrial membranes (OMM and IMM, respectively) is linked to alterations of redox state, IMM potential and bioenergetic efficiency [2–4]. This leads to indirect modulation of oxidative phosphorylation, which is fundamental for both cancer and cancer stem cell survival [5, 6]. Furthermore, inhibition of IMM channels, such as mitochondrial Kv1.3 [7] and TASK-3 [8] can cause an increase in ROS release, opening of the permeability transition pore (PTP) and trigger cytochrome c release leading to apoptosis [9]. These findings suggest a role for these channels in chemo-resistance [10]. Other ion channels of the IMM, such as the mitochondrial calcium uniporter also modulate cancer cell behavior in a ROS-dependent way and its silencing hampers tumor growth and metastatic spread [11]. In the outer membrane, the voltage-dependent anion channels (VDACs) regulate cancer cell metabolism and susceptibility to apoptotic stimuli [12, 13]. As most of these channels are differentially expressed and/or regulated in cancerous compared to non-malignant cells, their pharmacological targeting may selectively eliminate malignant cells [14].

The intermediate-conductance potassium channel K_Ca_3.1 (KCNN4) is highly expressed in many solid, leukemic and lymphatic cancer cells, whose proliferation it drives [15–17]. K_Ca_3.1 shows high expression also in pancreatic ductal adenocarcinoma (PDAC) [18], melanoma [19], triple-negative breast cancer (TNBC) [20] and non-small cell lung cancer [21]. To explore the role of KCa3.1 in tumor cell behavior most studies exploited inhibitors of the channel, while for TNBC the relevance of K_Ca_3.1 for tumorigenesis was elegantly confirmed using K_Ca_3.1^−/−^ mice [22]. K_Ca_3.1 has been identified as functional channel not only in the plasma membrane (PM) but also in the IMM of colon cancer, melanoma, HeLa, PDAC, and non-small cell lung cancer cells [18, 19, 21, 23, 24]. However, its pathophysiological impact on organelle function still awaits clarification.

The most potent small-molecule inhibitors of K_Ca_3.1 are TRAM-34 (1-[(2-Chlorophenyl)diphenylmethyl]-1H-pyrazole) [25] and Senicapoc (2,2-Bis(4-fluorophenyl)-2-phenylacetamide) [26], which selectively block the channel in electrophysiological experiments. The binding of TRAM-34 in K_Ca_3.1 has been modeled using the structure of KcsA [27]. TRAM-34 has been shown to be highly efficient against a variety of diseases such as auto-immune, inflammatory and cardiovascular disorders as well as a few types of cancer (for a recent review see e.g., [28]), while Senicapoc has entered clinical trial against sickle-cell disease [29]. TRAM-34 was found to reduce cancer cell proliferation [30–32] and, in a few cell types, to trigger apoptosis when used in combination with chemotherapeutics (e.g., [19]). On the other hand, activation of K_Ca_3.1 in combination with Kv1.11 blockers contrasted cisplatin resistance in colorectal cancer cells [33].

We aimed to dissect the specific role of the mitochondrial K_Ca_3.1 channel in tumor cell physiology by using two novel mitochondria-targeted derivatives of TRAM-34, designed following the strategy previously exploited by us for other mitochondrial ion channels [7, 8]. Here we show that very low, non-toxic concentrations of the TRAM-34 derivatives, in contrast to a membrane-impermeant K_Ca_3.1 toxin inhibitor, were able to reduce migration and colony formation by B16F10 melanoma and MDA-MB-231 triple-negative breast cancer lines, by affecting specific mitochondria-related pathways. At higher concentrations, both drugs triggered cell death when used on PDAC, melanoma and TNBC lines. These in vitro data were corroborated in vivo using two independent orthotopic models, in which a benefit was observed in terms of both tumor size and metastatic spread.

## Materials/subjects and methods

### Synthesis of TRAM-34 derivatives

HPLC grade acetonitrile (CH_3_CN) was obtained from Carlo Erba and used without further purification. Other reagents and solvents were purchased from Sigma-Aldrich and were used as received. Flash column chromatography was performed on silica gel (Macherey-Nagel 60, 230-400 mesh granulometry (0.063–0.040 mm)) under nitrogen pressure. ^1^H and ^13^C NMR spectra were recorded with a Bruker 500 Avance III operating at 500 MHz (for ^1^H NMR) and 126 MHz (for ^13^C NMR) or with a Bruker 400 Avance III HD operating at 400/101 MHz. Chemical shifts (δ) are given in parts per million (ppm) relative to the signal of the solvent. The following abbreviations are used to indicate multiplicities: s, singlet; d, doublet; dd, doublet of doublets; t, triplet; m, multiplet; p, pentet; br, broad signal. Electron spray ionization-mass spectrometry (ESI-MS) analysis was carried out with a 1100 Series Agilent Technologies system. The ESI source operated in full-scan positive ion mode, applying the following ESI parameters: nebulizer pressure 20 psi, dry gas flow 5 l/min, dry gas temperature 325 °C. The flow rate was 0.05 mL/min.

### Synthesis of mitoTRAM-34

#### Synthesis of 3-(4-bromophenyl)propan-1-ol (**1**)

A 1.0 M LiAlH_4_ solution in THF (45.4 mL, 45.4 mmol, 1.5 eq) was added dropwise to a solution of 3-(4-bromophenyl)propanoic acid (6.93 g, 30.2 mmol, 1.0 eq) in Et_2_O (150 mL) at 0 °C. The resulting gray suspension was stirred at 0 °C for few minutes, then at room temperature for 3.5 h under N_2_. HCl 1.0 M (150 mL) was carefully added to the suspension and the mixture was extracted with EtOAc (150 mL, 2 × 70 mL). The combined organic layers were washed with saturated solution of NaHCO_3_/brine 1:1 (2 × 200 mL), dried over Na_2_SO_4_ and concentrated under reduced pressure. The crude product was purified by flash chromatography (PE/EtOAc 65:35 as eluent) to afford **1** (6.11 g, 28.4 mmol, 94% yield). ^1^H NMR (500 MHz, CDCl_3_) δ 7.42–7.36 (m, 2H), 7.09–7.02 (m, 2H), 3.67–3.57 (m, 2H), 3.09–3.04 (m, 1H), 2.67–2.61 (m, 2H), 1.88–1.80 (m, 2H). ^13^C NMR (126 MHz, CDCl_3_) δ 140.75, 131.34, 130.16, 119.47, 61.62, 33.88, 31.37.

#### Synthesis of 1-(3-(benzyloxy)propyl)-4-bromobenzene (**2**)

A NaH 60% dispersion in mineral oil (1.36 g, 34.1 mmol, 1.2 eq) was added to a solution of **1** (6.11 g, 28.4 mmol, 1.0 eq) in dry DMF (40 mL). The mixture was stirred at 0 °C for 1 h under N_2_. Then, benzyl bromide (4.1 mL, 34 mmol, 1.2 eq) was added dropwise at 0 °C and the reaction mixture was stirred at room temperature for 3.5 h under N_2_. The reaction was quenched with water (30 mL) at 0 °C. The mixture was diluted with Et_2_O (100 mL) and washed with water (3 × 100 mL). The aqueous phase was extracted with Et_2_O (150 mL). The combined organic layers were dried over Na_2_SO_4_ and concentrated under reduced pressure. The crude product was purified by flash chromatography (PE/EtOAc 97:3 as eluent) to afford **2** (8.13 g, 26.6 mmol, 94% yield). ^1^H NMR (500 MHz, CDCl_3_) δ 7.46–7.39 (m, 6H), 7.38–7.33 (m, 1H), 7.11–7.07 (m, 2H), 4.56 (s, 2H), 3.52 (t, *J* = 6.2 Hz, 2H), 2.73 (t, 2H), 2.00–1.92 (m, 2H). ^13^C NMR (126 MHz, CDCl_3_) δ 140.96, 138.53, 131.39, 130.34, 128.45, 127.74, 127.65, 119.53, 72.99, 69.17, 31.83, 31.26.

#### Synthesis of (4-(3-(benzyloxy)propyl)phenyl)(2-chlorophenyl)(phenyl)methanol (**3**)

A 1.6 M n-butyllithium solution in hexanes (1.13 mL, 1.80 mmol, 1.2 eq) was added dropwise to a solution of **2** (500 mg, 1.64 mmol, 1.1 eq) in dry THF (4.1 mL) at −70 °C. The mixture was stirred under Ar for 1 h, after which the temperature reached −60 °C. Then the mixture was cooled down to −70 °C and 2-chlorobenzophenone (319 mg, 1.47 mmol, 1.0 eq) in THF (0.6 mL) was added dropwise. The mixture was stirred overnight and allowed to reach room temperature. The reaction was quenched with 0.5 M HCl (80 mL) and the mixture was extracted with EtOAc (3 × 80 mL). The combined organic layers were dried over MgSO_4_ and concentrated under reduced pressure. The crude product was purified by flash chromatography (starting eluent: PE/CHCl_3_ 7:3; final eluent: PE/Et_2_O 85:15) to afford **3** (521 mg, 1.18 mmol, 80% yield) as a colorless oil. ^1^H NMR (500 MHz, CDCl_3_) δ 7.44 (dd, *J* = 7.9, 1.2 Hz, 1H), 7.42–7.31 (m, 10H), 7.28 (td, *J* = 7.7, 1.6 Hz, 1H), 7.24–7.17 (m, 4H), 7.15 (td, *J* = 7.7, 1.2 Hz, 1H), 6.80 (dd, *J* = 7.9, 1.6 Hz, 1H), 4.58 (s, 2H), 3.57 (t, *J* = 6.4 Hz, 2H), 2.81–2.76 (m, 2H), 2.22 (s, 1H), 2.06–1.98 (m, 2H). ^13^C NMR (126 MHz, CDCl_3_) δ 145.69, 143.84, 142.98, 141.02, 138.27, 133.25, 131.50, 131.36, 129.11, 128.45, 128.13, 128.02, 127.84, 127.82, 127.75, 127.70, 127.38, 126.42, 82.58, 72.91, 69.48, 31.94, 31.12. ESI-MS (*m*/*z*): 425 [M-OH]^+^, 465 [M + Na]^+^, 481 [M + K]^+^.

#### Synthesis of 1-((4-(3-(benzyloxy)propyl)phenyl)(2-chlorophenyl)(phenyl)methyl)-1H-pyrazole (**5**)

Acetyl chloride (112 µL, 1.58 mmol, 3.5 eq) was added dropwise to a solution of **3** (200 mg, 0.451 mmol, 1.0 eq) in dry toluene (2.5 mL) and the mixture was stirred under reflux for 45 min. The solvent was removed under reduced pressure and the residue was dissolved in CH_3_CN (10.0 mL). Pyrazole (100 mg, 1.47 mmol, 3.3 eq) was added and the solution was stirred under reflux overnight, under Ar. The mixture was concentrated under reduced pressure and the crude product was purified by flash chromatography (PE/EtOAc 85:15 as eluent) to afford **5** (184 mg, 0.373 mmol, 83% yield) as a viscous colorless oil. ^1^H NMR (400 MHz, CDCl_3_) δ 7.69–7.63 (m, 1H), 7.50–7.45 (m, 1H), 7.44–7.39 (m, 1H), 7.38–7.15 (m, 12H), 7.15–7.04 (m, 4H), 6.93–6.87 (m, 1H), 6.29–6.23 (m, 1H), 4.51 (s, 2H), 3.50 (t, *J* = 6.3 Hz, 2H), 2.79–2.66 (m, 2H), 2.01–1.88 (m, 2H). ^13^C NMR (101 MHz, CDCl_3_) δ 142.00, 141.63, 141.59, 139.72, 139.22, 138.67, 135.93, 132.14, 131.89, 131.52, 130.36, 129.55, 128.50, 127.90, 127.79, 127.67, 126.84, 104.55, 78.25, 73.03, 69.59, 31.99, 31.13. ESI-MS (*m*/*z*): 425 [M-C_3_H_3_N_2_]^+^, 493 [M + H]^+^, 515 [M + Na]^+^, 531 [M + K]^+^.

#### Synthesis of 3-(4-((2-chlorophenyl)(phenyl)(1H-pyrazol-1-yl)methyl)phenyl)propan-1-ol) (**6**)

Pd/C (10 wt% loading, 18 mg) was added to a solution of **5** (184 mg, 0.373 mmol) in EtOAc (4.0 mL). The mixture was stirred at room temperature for 75 min under H_2_ atmosphere and, then, filtered through celite. After removal of the solvent under reduced pressure, the crude product was purified by flash chromatography (PE/EtOAc 6:4 as eluent) to afford **6** (135 mg, 0.335 mmol, 90% yield) as a foamy white solid. ^1^H NMR (500 MHz, CDCl_3_) δ 7.65 (d, *J* = 1.4 Hz, 1H), 7.48 (d, *J* = 2.3 Hz, 1H), 7.40 (dd, *J* = 7.9, 1.2 Hz, 1H), 7.33–7.27 (m, 4H), 7.23–7.16 (m, 3H), 7.15–7.06 (m, 4H), 6.90 (dd, *J* = 8.0, 1.5 Hz, 1H), 6.28–6.22 (m, 1H), 3.62 (t, *J* = 6.4 Hz, 2H), 2.72–2.65 (m, 2H), 1.91–1.83 (m, 2H). ^13^C NMR (126 MHz, CDCl_3_) δ 141.87, 141.47, 141.45, 139.66, 139.19, 135.81, 132.10, 131.82, 131.44, 130.34, 130.24, 129.51, 127.78, 127.72, 126.79, 104.52, 78.16, 62.13, 33.90, 31.62. ESI-MS (*m*/*z*): 335 [M-C_3_H_3_N_2_]^+^, 425 [M + Na]^+^, 441 [M + K]^+^.

#### Synthesis of (1-((2-chlorophenyl)(4-(3-chloropropyl)phenyl)(phenyl)methyl)-1H-pyrazole) (**7**)

LiCl (1.19 g, 28.1 mmol, 15 eq), 2,4,6-trimethylpyridine (1.48 mL, 11.2 mmol, 6.0 eq), and methanesulfonyl chloride (724 µL, 9.34 mmol, 5.0 eq) were added to a solution of **6** (754 mg, 1.87 mmol, 1.0 eq) in dry DMF (20 mL). The mixture was stirred for 15 h at room temperature, then it was diluted with EtOAc (200 mL) and washed with brine/0.5 M HCl 1:1 (2 × 300 mL) and brine/H_2_O 1:1 (2 × 300 mL). The organic phase was dried over Na_2_SO_4_ and concentrated under reduced pressure. The crude product was purified by flash chromatography (PE/EtOAc 9:1 as eluent) to afford **7** (699 mg, 1.66 mmol, 89% yield). ^1^H NMR (500 MHz, CDCl_3_) δ 7.66 (d, *J* = 1.6 Hz, 1H), 7.47 (d, *J* = 2.4 Hz, 1H), 7.41 (dd, *J* = 7.8, 1.3 Hz, 1H), 7.35–7.27 (m, 4H), 7.22 (td, *J* = 7.9, 1.3 Hz, 1H), 7.20–7.16 (m, 2H), 7.16–7.08 (m, 4H), 6.90 (dd, *J* = 8.0, 1.5 Hz, 1H), 6.28–6.25 (m, 1H), 3.54 (t, *J* = 6.5 Hz, 2H), 2.82–2.74 (m, 2H), 2.14–2.05 (m, 2H). ^13^C NMR (126 MHz, CDCl_3_) δ 141.91, 141.52, 140.27, 139.76, 139.65, 135.88, 132.15, 131.91, 131.50, 130.52, 130.29, 129.61, 127.94, 127.82, 126.87, 104.60, 78.20, 44.39, 33.82, 32.40. ESI-MS (*m*/*z*): 353 [M-C_3_H_3_N_2_]^+^, 443 [M + Na]^+^, 459 [M + K]^+^.

#### Synthesis of (3-(4-((2-chlorophenyl)(phenyl)(1H-pyrazol-1yl)methyl)phenyl)propyl)triphenylphosphonium iodide (mitoTRAM-34)

Triphenylphosphine (5.66 g, 21.6 mmol, 13 eq) and NaI (2.49 g, 16.6 mmol, 10 eq) were added to a solution of **7** (699 mg, 1.66 mmol, 1.0 eq) in CH_3_CN (7.0 mL). The mixture was stirred in a sealed vial for 6 h at 95 °C. H_2_O (100 mL) was then added and the mixture was extracted with DCM (3 × 100 mL). The combined organic layers were dried over MgSO_4_ and the solvent was removed under reduced pressure. The crude product was purified by flash chromatography (starting eluent: CHCl_3_; final eluent: CHCl_3_/MeOH 98:2) to afford mitoTRAM (1.04 g, 1.34 mmol, 81% yield) as a foamy white solid. ^1^H NMR (500 MHz, CDCl_3_) δ 7.78–7.67 (m, 9H), 7.67–7.61 (m, 6H), 7.59 (d, *J* = 1.4 Hz, 1H), 7.39 (d, *J* = 2.5 Hz, 1H), 7.36 (dd, *J* = 7.9, 1.4 Hz, 1H), 7.32–7.23 (m, 4H), 7.19 (td, *J* = 7.7, 1.5 Hz, 1H), 7.14–7.04 (m, 6H), 6.82 (dd, *J* = 8.0, 1.5 Hz, 1H), 6.25–6.21 (m, 1H), 3.76–3.59 (m, 2H), 3.02 (t, *J* = 7.2 Hz, 2H), 2.01–1.88 (m, 2H). ^13^C NMR (126 MHz, CDCl_3_) δ 141.81, 141.36, 139.94, 139.65, 139.52, 135.66, 135.20 (d, *J* = 2.7 Hz), 133.74 (d, *J* = 10.0 Hz), 132.26, 131.88, 131.51, 130.63 (d, *J* = 12.6 Hz), 130.54, 130.06, 129.72, 128.29, 127.82, 126.93, 118.03 (d, *J* = 86.1 Hz), 104.62, 78.09, 35.14 (d, *J* = 16.6 Hz), 24.28 (d, *J* = 3.3 Hz), 22.03 (d, *J* = 50.9 Hz). ESI-MS (*m*/*z*): 647.2 [M-I]^+^.

### Synthesis of intermediate 8

#### Synthesis of 4-nitrophenyl (3-chloropropyl)carbamate (**8**)

3-chloropropylamine hydrochloride (2.60 g, 20.0 mmol, 1.0 eq) was added to a solution of DMAP (4.89 g, 40.0 mmol, 2.0 eq) in DCM (85 mL). The resulting solution was added dropwise to a stirred solution of bis(4-nitrophenyl) carbonate (6.69 g, 22.0 mmol, 1.1 eq) in THF (170 mL). After 3.5 h at room temperature the solvent was removed under reduced pressure. EtOAc (500 mL) was added and the mixture was washed with 0.5 M HCl (500 mL). The organic phase was dried over Na_2_SO_4_ and the solvent was removed under reduced pressure. The crude product was purified by flash chromatography (DCM/EtOAc 98:2 as eluent) to afford **8** (4.54 g, 17.6 mmol, 88% yield) as a white solid. ^1^H NMR (500 MHz, CDCl_3_) δ 8.27–8.20 (m, 2H), 7.35–7.28 (m, 2H), 5.49–5.34 (m, 1H), 3.64 (t, *J* = 6.2 Hz, 2H), 3.46 (q, *J* = 6.5 Hz, 2H), 2.07 (p, *J* = 6.5 Hz, 2H). ^13^C NMR (126 MHz, CDCl_3_) δ 155.93, 153.34, 144.86, 125.22, 122.06, 42.23, 38.87, 32.09. ESI-MS (*m*/*z*): 259 [M + H]^+^.

### Synthesis of *rev*-mitoTRAM

#### Synthesis of 1-(benzyloxy)-3-bromobenzene (**9**)

K_2_CO_3_ (771 mg, 5.58 mmol, 3.0 eq) and benzyl bromide (351 mg, 2.05 mmol, 1.1 eq) were added to a solution of 3-bromophenol (322 mg, 1.86 mmol, 1.0 eq) in acetone (11.0 mL). The suspension was stirred in a sealed vial for 15 h at 70 °C, then it was filtered to remove the precipitate. The solvent was removed under reduced pressure and the crude product was purified by flash chromatography (starting eluent: PE; final eluent: PE/EtOAc 95:5) to afford **9** (452 mg, 1.72 mmol, 92% yield) as a white solid. ^1^H NMR (500 MHz, CDCl_3_) δ 7.48–7.40 (m, 4H), 7.40–7.35 (m, 1H), 7.20–7.18 (m, 1H), 7.17 (d, *J* = 8.0 Hz, 1H), 7.15–7.12 (m, 1H), 6.96–6.92 (m, 1H), 5.06 (s, 2H). ^13^C NMR (126 MHz, CDCl_3_) δ 159.64, 136.49, 130.68, 128.75, 128.26, 127.60, 124.17, 122.93, 118.28, 113.92, 70.28.

#### Synthesis of (3-(benzyloxy)phenyl)(2-chlorophenyl)(phenyl)methanol (**10**)

A 2.5 M n-butyllithium solution in hexanes (746 µL, 1.87 mmol, 1.2 eq) was added dropwise to a solution of **9** (450 mg, 1.71 mmol, 1.1 eq) in dry THF (4.5 mL) at −70 °C. The mixture was stirred under Ar for 1 h, after which the temperature reached −60 °C. Then the mixture was cooled down to −70 °C and 2-chlorobenzophenone (337 mg, 1.55 mmol, 1.0 eq) in THF (1.0 mL) was added dropwise. The mixture was stirred for 16 h and allowed to reach room temperature. The reaction was quenched with 0.5 M HCl (50 mL) and the mixture was extracted with DCM (50 mL, 3 × 30 mL). The combined organic layers were dried over MgSO_4_ and concentrated under reduced pressure. The crude product was purified by flash chromatography (PE/acetone 97:3 as eluent) to afford **10** (312 mg, 0.778 mmol, 50% yield) as a viscous transparent oil. ^1^H NMR (500 MHz, CDCl_3_) δ 7.43–7.25 (m, 13H), 7.14 (td, *J* = 7.7, 1.3 Hz, 1H), 7.02–6.99 (m, 1H), 6.98–6.94 (m, 1H), 6.87–6.83 (m, 1H), 6.74 (dd, *J* = 7.9, 1.6 Hz, 1H), 5.04 (s, 2H), 4.47 (s, 1H). ^13^C NMR (126 MHz, CDCl_3_) δ 158.72, 147.36, 145.45, 143.72, 136.99, 133.30, 131.62, 131.45, 129.23, 129.08, 128.66, 128.13, 128.06, 127.84, 127.77, 127.54, 126.53, 120.72, 114.63, 114.00, 82.65, 70.08. ESI-MS (*m*/*z*): 423 [M + Na]^+^.

#### Synthesis of 1-((3-(benzyloxy)phenyl)(2-chlorophenyl)(phenyl)methyl)-1H-pyrazole (**12**)

Acetyl chloride (166 µL, 2.33 mmol, 3.0 eq) was added dropwise to a solution of **10** (312 mg, 0.778 mmol, 1.0 eq) in dry toluene (5.0 mL) and the mixture was stirred in a sealed vial for 1.5 h at 100 °C. The solvent was removed under reduced pressure and the residue was dissolved in CH_3_CN (14.0 mL). Pyrazole (159 mg, 2.33 mmol, 3.0 eq) was added and the solution was stirred in the sealed vial for 17 h at 100 °C. The mixture was concentrated under reduced pressure and the crude product was purified by flash chromatography (PE/EtOAc 9:1 as eluent) to afford **12** (238 mg, 0.528 mmol, 68% yield) as a viscous transparent oil. ^1^H NMR (500 MHz, CDCl_3_) δ 7.72–7.69 (m, 1H), 7.53–7.51 (m, 1H), 7.43 (dd, *J* = 7.9, 1.4 Hz, 1H), 7.39–7.30 (m, 9H), 7.26–7.20 (m, 4H), 6.99–6.95 (m, 2H), 6.94 (t, *J* = 2.1 Hz, 1H), 6.81–6.77 (m, 1H), 6.30–6.28 (m, 1H), 4.98 (s, 2H). ^13^C NMR (126 MHz, CDCl_3_) δ 158.26, 143.58, 141.65, 141.23, 139.71, 136.80, 135.90, 132.17, 131.83, 131.57, 130.17, 129.56, 128.60, 128.55, 127.95, 127.74, 127.63, 126.75, 123.05, 117.26, 114.11, 104.51, 78.20, 69.96. ESI-MS (*m*/*z*): 383 [M-C_3_H_3_N_2_]^+^, 473 [M + Na]^+^, 489 [M + K]^+^.

#### Synthesis of 3-((2-chlorophenyl)(phenyl)(1H-pyrazol-1-yl)methyl)phenol (TRAM-34-OH)

Pd/C (10 wt% loading, 26 mg) was added to a solution of **12** (238 mg, 0.528 mmol) in EtOAc (6.0 mL). The mixture was stirred for 1.5 h under H_2_ atmosphere and, then, filtered through celite. After removal of the solvent under reduced pressure, the crude product was purified by flash chromatography (PE/EtOAc 8:2 as eluent) to afford TRAM-OH (166 mg, 0.459 mmol, 87% yield) as a foamy white solid. ^1^H NMR (500 MHz, CDCl_3_) δ 7.55 (d, *J* = 2.5 Hz, 1H), 7.53 (d, *J* = 1.5 Hz, 1H), 7.39 (dd, *J* = 7.9, 1.3 Hz, 1H), 7.32–7.26 (m, 4H), 7.22–7.15 (m, 3H), 7.06 (t, *J* = 8.0 Hz, 1H), 6.96 (dd, *J* = 8.0, 1.5 Hz, 1H), 6.72–6.68 (m, 1H), 6.63–6.56 (m, 2H), 6.56–6.51 (m, 1H), 6.25–6.22 (m, 1H). ^13^C NMR (126 MHz, CDCl_3_) δ 156.08, 143.51, 141.38, 140.99, 139.82, 135.98, 132.50, 131.95, 131.84, 130.37, 129.71, 128.68, 127.95, 127.86, 126.85, 122.39, 117.33, 115.06, 104.67, 78.40. ESI-MS (*m*/*z*): 293 [M-C_3_H_3_N_2_]^+^, 383 [M + Na]^+^.

#### Synthesis of 3-((2-chlorophenyl)(phenyl)(1H-pyrazol-1-yl)methyl)phenyl (3-chloropropyl)carbamate (**13**)

**8** (86.9 mg, 336 µmol, 2.0 eq) and DMAP (41.0 mg, 336 µmol, 2.0 eq) were added to a solution of TRAM-OH (60.6 mg, 168 µmol, 1.0 eq) in CH_3_CN (7.0 mL). The mixture was stirred in a sealed vial for 20 h at 50 °C, then it was concentrated under reduced pressure and DCM (25 mL) was added. The mixture was washed with 0.5 M HCl (25 mL) and the aqueous phase was extracted with DCM (2 × 25 mL). The combined organic layers were dried over Na_2_SO_4_ and the solvent was removed under reduced pressure. The crude product was purified by flash chromatography (DCM/EtOAc 95:5 as eluent) to afford **13** (66.0 mg, 137 µmol, 82% yield) as a foamy white solid. ^1^H NMR (500 MHz, CDCl_3_) δ 7.65 (d, *J* = 1.4 Hz, 1H), 7.52 (d, *J* = 2.3 Hz, 1H), 7.41 (dd, *J* = 7.9, 1.3 Hz, 1H), 7.34–7.27 (m, 5H), 7.24–7.20 (m, 1H), 7.20–7.15 (m, 2H), 7.15–7.11 (m, 1H), 7.03–6.96 (m, 2H), 6.93 (dd, *J* = 8.0, 1.5 Hz, 1H), 6.28–6.26 (m, 1H), 5.28 (t, *J* = 6.0 Hz, 1H), 3.57 (t, *J* = 6.3 Hz, 2H), 3.38–3.32 (m, 2H), 1.99 (p, *J* = 6.5 Hz, 2H). ^13^C NMR (126 MHz, CDCl_3_) δ 154.60, 150.56, 143.42, 141.48, 141.00, 139.87, 135.85, 132.32, 131.95, 131.62, 130.22, 129.76, 128.46, 127.94, 127.90, 127.19, 126.94, 123.61, 121.07, 104.76, 78.04, 42.30, 38.62, 32.24. ESI-MS (*m*/*z*): 412 [M-C_3_H_3_N_2_]^+^, 502 [M + Na]^+^, 518 [M + K]^+^.

#### Synthesis of (3-(((3-((2-chlorophenyl)(phenyl)(1H-pyrazol-1-yl)methyl)phenoxy) carbonyl)amino)propyl) triphenylphosphonium iodide (rev-mitoTRAM)

Triphenylphosphine (180 mg, 685 μmol, 5.0 eq) and NaI (205 mg, 1.37 mmol, 10 eq) were added to a solution of **13** (66.0 mg, 137 μmol, 1.0 eq) in CH_3_CN (2.0 mL). The mixture was stirred in a sealed vial for 6 h at 95 °C. H_2_O (25 mL) was then added and the mixture was extracted with DCM (3 × 25 mL). The combined organic layers were dried over Na_2_SO_4_ and the solvent was removed under reduced pressure. The crude product was purified two times by flash chromatography (first column starting eluent: CHCl_3_, final eluent: CHCl_3_/MeOH 95:5; second column starting eluent: CHCl_3_/MeOH 98:2, final eluent: CHCl_3_/MeOH 96:4) to afford mitoTRAM-OH (82.6 mg, 99.0 μmol, 72% yield) as a foamy off-white solid. ^1^H NMR (500 MHz, CDCl_3_) δ 7.76–7.68 (m, 9H), 7.66–7.60 (m, 6H), 7.56 (d, *J* = 1.5 Hz, 1H), 7.47 (d, *J* = 2.4 Hz, 1H), 7.35 (dd, *J* = 7.9, 1.3 Hz, 1H), 7.29–7.22 (m, 4H), 7.22–7.14 (m, 2H), 7.13–7.07 (m, 2H), 7.05–7.01 (m, 1H), 6.98–6.92 (m, 3H), 6.85 (dd, *J* = 8.0, 1.4 Hz, 1H), 6.21–6.19 (m, 1H), 3.71–3.61 (m, 2H), 3.52–3.44 (m, 2H), 1.93–1.83 (m, 2H). ^13^C NMR (126 MHz, CDCl_3_) δ 154.83, 150.65, 143.05, 141.55, 140.96, 139.67, 135.71, 135.26 (d, *J* = 2.5 Hz), 133.53 (d, *J* = 10.0 Hz), 132.19, 131.84, 131.50, 130.64 (d, *J* = 12.6 Hz), 130.11, 129.66, 128.18, 127.78, 126.83, 126.78, 123.80, 121.15, 117.90 (d, *J* = 86.4 Hz), 104.64, 77.95, 40.35 (d, *J* = 17.7 Hz), 22.66 (d, *J* = 3.2 Hz), 21.13 (d, *J* = 52.4 Hz). ESI-MS (*m*/*z*): 706.3 [M-I]^+^.

### Other chemical compounds

All powders and commercially available compounds (including TRAM-34, staurosporin, oligomycin, FCCP and antimycin A) were from Sigma-Aldrich (Milan, Italy) and were solved in DMSO at appropriate stock solution concentrations (at least 1000X).

### Docking analysis

The molecular docking calculations were performed on two K_Ca_3.1 homology models taken from [27] using three different molecules: TRAM-34, TRAM-34-OH, and mitoTRAM-34. First, we prepared our channel/ligand .pdbqt files by employing the ADFR suite [34, 35]. Next, we used AutoDock [36] to prepare our configuration file. Finally, the docking was performed using AutoDock Vina [37] by completely encapsulating channels in grid boxes with XYZ size 44 × 66 × 64 Å^3^ (K_Ca_3.1/Kv1.2) or 44 × 44 × 46 Å^3^ (K_Ca_3.1/KcsA).

### Cell culture

Cells were maintained in a humidified incubator at 37 °C and 5% CO_2_, passed twice a week and regularly checked for mycoplasma infection. B16F10 cells were grown in MEM (Gibco) supplemented with 10% (v/v) fetal bovine serum (FBS, Gibco), 100 U/mL penicillin G, 0.1 mg/mL streptomycin (Gibco), 1% non-essential amino acids (NEAA, 100X solution, Gibco), 2 mM L-Glutamine (100X solution, Gibco) and 1 mM sodium pyruvate (100X solution, Gibco). GL-15 cells were cultured in DMEM (4.5 g/L D-glucose) supplemented with 10% FBS, 100 U/mL penicillin G, and 0.1 mg/mL streptomycin. Melan-A cells (obtained from the Wellcome Trust Functional Genomics Cell Bank at St. George’s, University of London [38]) were cultured in RPMI-1640 supplemented with 10% FBS, 100 U/ml penicillin G, 0.1 mg/mL streptomycin, 2 mM L-Glutamine and 200 nM phorbol 12-myristate 13-acetate (Sigma-Aldrich) freshly added at each passage. MDA-MB-231 human triple-negative breast cancer cells were cultured in DMEM-F12 (Gibco) supplemented with 10% (v/v) FBS, 100 U/mL penicillin G, 0.1 mg/mL streptomycin, 1% non-essential amino acids, 2 mM L-Glutamine and 1 mM sodium pyruvate. HEK-293, MIA PaCa-2 and PANC-1 cells were cultured in DMEM (4.5 g/L D-glucose) supplemented with 10% FBS, 100 U/mL penicillin G, 0.1 mg/mL streptomycin, 10 mM Hepes (100X solution, Gibco) and 1% NEAA. PAN-02 cells were cultured in DMEM (4.5 g/L D-glucose) supplemented with 10% FBS. COLO-357 cells were cultured in RPMI-1640 (Gibco) supplemented with 10% FBS, 100 U/ml penicillin G, 0.1 mg/mL streptomycin, 10 mM Hepes and 1% NEAA. HPDE cells were cultured in 50% RPMI + 50% Keratinocyte-SFM (serum-free medium, Gibco) supplemented with 10% FBS, 10 mM HEPES, 100 U/ml penicillin G, 0.1 mg/mL streptomycin, 1X NEAA, 2.5 µg/L human recombinant epidermal growth factor (EGF, Gibco) and 0.025% bovine pituitary extract (BPE, Gibco) freshly added at each passage. MMTV-PyMT primary breast cancer cells from WT of KCa3.1-KO mice [20] were cultured in IMEM (Gibco) supplemented with 5% FBS, 100 U/mL penicillin G, and 0.1 mg/mL streptomycin.

### Analysis of the hydrolysis of *rev*-mitoTRAM

B16F10 cells were seeded in 100 mm culture dishes and allowed to grow to 80–90% confluence. Cells were treated for 2, 4, 6, 8, or 24 h with 5 µM rev-mitoTRAM-34 in 8 ml phenol red- and serum-free medium. The medium was then removed, cells were washed with PBS, harvested in 2 ml ice-cold PBS with a scraper and centrifuged at 800 × *g* for 5 min at 4 °C. The medium and the cell pellet were stored at −80 °C until analysis. Cell pellets were resuspended in 100 μl acetonitrile (CH_3_CN) with 0.1% trifluoroacetic acid (TFA), vortexed, sonicated for 5 minutes and centrifuged at 12,000 × *g* for 7 min. The supernatant was collected and analyzed by HPLC-UV. The medium was centrifuged (12,000 × *g*, 7 min) to remove cell debris, and analyzed without further treatments. HPLC analysis was performed with a 1290 Infinity LC System (Agilent Technologies) equipped with a UV diode array detector (190–500 nm), using a reverse phase column (Zorbax RRHT Extend-C18, 1.8 μm, 50 × 3.0 mm i.d., Agilent Technologies) kept at 35 °C. Solvents A and B were water containing 0.1% TFA and acetonitrile, respectively. The gradient for solvent B was as follows: 10% for 0.5 min, then from 10% to 100% in 4.5 min, 100% for 1 min. The flow rate was 0.6 mL/min. The eluate was preferentially monitored at 270 nm. Analytes were quantified using a calibration curve correlating the peak area with the concentration of the analytes.

### Electrophysiological recordings

For electrophysiological experiments, 30,000 human glioblastoma cells (GL-15 cell line) were seeded in 35 mm Petri dishes and the experiments were conducted in the same dish 2–3 days after plating. The patch-clamp recording was performed in whole-cell perforated patch-clamp configuration [39, 40]. Currents and voltages were amplified with a HEKA EPC-10 amplifier and analyzed with the PatchMaster and Origin 4.1 softwares. For online data collection, currents were filtered at 3 kHz, and sampled at 100 µs/point. During recordings the extracellular solution had the following composition (mM): NaCl 106.5, KCl 5, CaCl_2_ 2, MgCl_2_ 2, MOPS 5, Glucose 20, Na-gluconate 30 (pH 7.25 with NaOH). The intracellular solution was (mM) K_2_SO_4_ 57.5, KCl 55, MgCl_2_ 5, MOPS 10, Glucose 20 (pH 7.2 with KOH). Electrical access to the cytoplasm was achieved by adding amphotericin B (200 µM) to the intracellular solution [39]. 1 mM octanol, a gap junction blocker, and 3 mM TEA, a BK_Ca_ blocker that does not block K_Ca_3.1 [39], were added to all external solutions to avoid interferences of gap junctions and BK_Ca_ currents, respectively [39, 40].

### Isolation of mitochondria from cultured cells

B16F10 cells were seeded in 150 mm culture dishes in normal culture medium. About 60*10^6^ cells at 80–90% confluency were harvested with a scraper, washed in ice-cold PBS and resuspended in TES buffer (300 mM sucrose, 10 mM TES, 0.5 mM EGTA, pH 7.4). Resuspended cells were kept on ice for 1 h, then fragmented with a Dounce homogenizer. Intact cells were pelleted by centrifugation at 1000 × *g* for 10 min at 4 °C, resuspended in TES buffer, homogenized and centrifuged again. The supernatants were pooled (200 µl were stored at −80 °C as “fraction 1”) and centrifuged at 6000 × *g* for 10 min at 4 °C. The resulting pellet was resuspended in a small volume of TES buffer (50 µl were stored at −80 °C as “fraction 2”) and further purified on a Percoll gradient (60%, 30%, and 18% in TES buffer) by centrifugation at 8500 × *g* for 10 min at 4 °C. The floating material in the 18% layer, containing ER and plasma membrane contaminants (denominated “fraction 3”), at the upper interface, containing purified mitochondria (denominated “m1”) and at the lower interface (between the 30% and 18% fraction), containing further purified mitochondria (denominated “m2”), was collected and washed three times in TES buffer with centrifugation at 17,000 × *g* for 10 min at 4 °C. The final pellet was resuspended in buffer and stored at −80 °C.

### Western blotting

For mitochondrial isolation experiments, 25 µg protein were loaded on 4–20% Bis-Tris gels (Genscript). Proteins were blotted on polyvinylidene difluoride (PVDF) membranes with BioRad Transblot® Turbo™ standard protocols. Membranes were blocked in 2% BSA in TBS for 1 h at room temperature (RT) and incubated overnight with one of the following antibodies: PMCA (1:2000 in TTBS, Thermo Fisher Scientific #MA3-914); K_Ca_3.1 (Alomone Labs #APC-064); VDAC-1 (1:2000 in TTBS, Santa Cruz Biotechnologies (SCBT) #sc-390996); TOM-20 (1:1000 in TTBS, SCBT #sc-11415). Anti-mouse (1:5000, BioRad #1706516) or anti-rabbit (1:10,000, Jackson Immunoresearch #111-035-003) HRP-conjugated secondary antibodies were used for 1 h at RT in TTBS. Clarity Western ECL Substrate (BioRad) was used as chemiluminescence substrate. Signals were revealed using the ChemiDoc Gel Imaging System (BioRad).

To analyse changes in protein expression after treatment, 150,000 B16F10 cells were seeded in normal culture medium in 6-well plates, treated in phenol red-free DMEM with 0.5 µM mitoTRAM-34, 5 µM *rev*-mitoTRAM or the corresponding amount of DMSO (0.1%) for 24 h, washed in PBS and lysed in 25 mM Tris pH 7.8, 2.5 mM EDTA, 10% glycerol, 1% NP-40, 2 mM DTT, 1X protease and phosphatase inhibitors. 30 µg of each sample were loaded on precast gels and blotted as above. The membrane was incubated overnight with one of the following primary antibodies: α-AMPK pT172 (1:1000 in TBS-2% BSA, Cell Signaling Technologies (CST) #2535), α-HIF-1α (1:500 in TTBS, Genetex #GTX127309), α-LOXL-2 (1:1000 in TTBS, Genetex #GTX105085), α-Total OXPHOS Rodent WB Antibody Cocktail (1:3000 in TTBS, Abcam #ab110413), TOM-20 (1:1000 in TTBS, SCBT #sc-11415), α-BNIP-3 (1:1000 in TTBS, CST #3769), α-CDC-42 (1:500 in TTBS, Genetex #GTX134588), α-NFκB pS536 (1:500 in TTBS, CST #3033), α-NFκB (1:1000 in TTBS, CST #8242), α-vinculin (1:3000 in TTBS, EMD Millipore #AB6039), α-actin (1:5000 in TTBS, EMD Millipore #MAB1501). The signal was detected as described above.

### Mitochondrial membrane potential and ROS production

6,500 B16F10 cells/well were seeded in glass-bottom 96-well cell imaging plates in standard culture medium and incubated for 24 h. Then, cells were incubated with either 25 nM Tetramethylrhodamine (TMRM) (ThermoFisher) or 1 μM mitoSOX Red (ThermoFisher) in HBSS for 20 min at 37 °C. The medium was then substituted with HBSS (supplemented with 5 nM TMRM in the case of membrane potential determinations) and cells were imaged for 10 minutes using the Operetta system (Perkin Elmer). After 10 minutes, treatment compounds at the indicated concentration were added and cells were imaged every 5 min for 40 min. 5 fields/well were analyzed. Each condition was tested in duplicate. Analysis was performed using Harmony high-content analysis software.

### Electron microscopy

15,000 B16F10 cells were seeded in 24-w plates in standard culture medium and allowed to grow for 24 h. After treatment, performed as indicated in figure legends, cells were fixed for transmission electron microscopy (TEM) in a 2.5% (v/v) glutaraldehyde solution in 100 mM sodium cacodylate, pH 7.2, at 4 °C overnight. Following washing, post-fixation was performed in a 1% OsO_4_ solution in 100 mM sodium cacodylate, pH 7.2, at 4 °C. Sections were contrasted with a saturated uranyl acetate solution in 100% ethanol for 15 min, followed by incubation in a 1% (w/v) lead citrate solution in 100% ethanol for 7 min. Finally, the samples were observed with a Tecnai G2 Spirit transmission electron microscope (Fei electron microscopes) operating at 100 kV.

### Immunofluorescence analysis

For immunofluorescence analyses on cells, 15,000 B16F10 cells were seeded on 12 mm glass coverslips in 24-well plates. 24 h after seeding, cells were treated for 24 h with 0.5 µM mitoTRAM-34, 5 µM *rev*-mitoTRAM or the corresponding amount of DMSO (0.1%). For rescue experiments, cells were co-treated with 2.5 µg/ml Rho/Rac/Cdc42 Activator I (Cytoskeleton, Inc. #CN-04A). After treatment, cells were washed 1x in PBS, fixed in 3.8% PFA for 15 min, washed 3x in PBS, permeabilized for 10 min in 0.1% Triton X-100 (Sigma-Aldrich) in PBS, washed 3x in PBS, blocked for 1 h in 1% BSA + 5% goat serum in PBS and incubated overnight at 4 °C in 1% BSA + 5% goat serum in PBS with anti-TOM-20 (1:500, SCBT #sc-11415). The next day, cells were washed 3x in PBS and incubated for 1 h at room temperature with secondary antibody anti-rabbit AF488, ThermoFisher #A-11008, 1:1000 in 5% goat serum in PBS) or with 150 nM AF488 Phalloidin (ThermoFisher #A12379) in 5% goat serum in PBS. Samples were washed 3x in PBS and mounted with ProLong Gold Antifade Mountant with DAPI (Thermo Fisher Scientific) and images were acquired with a Zeiss LSM900 Airyscan2 confocal microscope.

### Oxygen consumption assay and activity of ATP synthase

Respiration was measured using an XFe24 Extracellular Flux Analyzer (SeaHorse), which measures the oxygen consumption rate (OCR). 30,000 B16F10 cells were seeded in standard culture medium and incubated for 24 h at 37 °C. The medium was then replaced with high-glucose DMEM without serum and supplemented with 1 mM sodium pyruvate and 4 mM L-glutamine. OCR was measured at pre-set time intervals upon the automated additions of the following compounds: treatment as indicated; 1 µg/mL oligomycin; 300 nM FCCP; 1 µM antimycin A.

The activity of ATP synthase was assayed in vitro using mouse liver mitochondria isolated following the protocol described in [41] or on crude cellular mitochondrial extracts (5*10^6^ cells were collected with a scraper, washed twice in PBS, resuspended in buffer containing 20 mM MOPS KOH pH 7.4, 250 mM sucrose, 0.1 mg/ml digitonin, incubated 5 min on ice and centrifuged at 5000 × *g* for 3 min. The pellet was resuspended in buffer containing 20 mM MOPS KOH pH 7.4, 250 mM sucrose, 1 mM Na-EDTA, incubated 5 min on ice, and centrifuged at 10,000 × *g* for 3 min. The pellet containing mitochondria was snap-frozen in liquid nitrogen and conserved at −80 °C until further use). Activities of complexes I and III were measured as described in [42].

Mitochondrial ATP-ase activity was measured by coupling the production of ADP (reverse mode) to the oxidation of NADH via the pyruvate kinase and lactate dehydrogenase reaction. 20 µg/mL mitochondria ± treatments were added to a reaction mixture (pH 7.4) containing 30 mM sucrose, 50 mM Tris–HCl, 2 mM EGTA, 50 mM KCl, 4 mM MgCl_2_, 4 mM phosphoenolpyruvate, pyruvate kinase (4 U/mL) and lactate dehydrogenase (3 U/mL). Absorbance was measured at 340 nm at 37 °C. The addition of 2 mM ATP and 0.2 mM NADH started the reaction. The absorbance of the sample treated with 1 µg/mL oligomycin A was subtracted to eliminate F_o_F_1_ ATP synthase-independent ATP hydrolysis. The activity was evaluated from the changes in the slope of the absorbance vs time plot; data are expressed as % of the control.

To analyse not ATP hydrolysis, but ATP synthesis, ATP production was followed over time in coupled, isolated mouse liver mitochondria. Briefly, 2 mg/ml mitochondria were resuspended in buffer (125 mM KCl, 10 mM Tris-MOPS, 1 mM EGTA, 2 mM K_2_HPO_4_ pH 7.4) containing 1 µM rotenone ± the different treatments as indicated in the figure. The reaction was started adding 5 mM succinate and 2 mM ADP and stopped at the indicated time points by adding the ATP lysis solution from the ATPlite Luminescence ATP Detection Assay System (Promega). Buffer containing mitochondria but not the starters for the reaction (succinate + ADP) was used as blank. Oligomycin-treated mitochondria were used as negative controls.

To check any possible effects on pyruvate kinase and lactate dehydrogenase activity (used in the ATP hydrolysis assay described above), a reaction mixture (pH 7.4) containing 30 mM sucrose, 50 mM Tris–HCl, 2 mM EGTA, 50 mM KCl, 4 mM MgCl_2_, 4 mM phosphoenolpyruvate, pyruvate kinase (1 U/mL), lactate dehydrogenase (1.5 U/mL) and 0.2 mM NADH ± treatments was prepared. Absorbance of NADH was measured at 340 nm at 37 °C over time and remained stable until the addition of 2 mM ADP as a starter of the reaction.

### MTS assay and calculation of EC_50_

For MTS assays, 5000–15,000 cells/well (according to the cell type) were seeded in standard 96-well plates and allowed to grow in standard culture medium for 24 h (70% confluency). The growth medium was then replaced with phenol red-free DMEM containing the treatment compound at the final concentration. Each condition was tested in quadruplicate. After treatment for 24 h, CellTiter 96 AQUEOUS One Solution (Promega) was used to determine cell viability following the manufacturer’s protocols. The percentage of viable cells compared to the control was plotted against log10 of the drug concentration and the concentration at which 50% of cells were viable was calculated using the sigmoidal curve fitting function of OriginLab.

### Annexin V assay

15,000 B16F10/well were seeded in standard 48-well plates and allowed to grow in normal culture medium for 24 h. The growth medium was then replaced with phenol red-free DMEM containing the treatment compound at the final concentration. After treatment for 24 h, 0.5 µl Annexin V-FLUOS (Roche) and 5 µg/mL Hoechst 33342 (Invitrogen) were directly added to each well, incubated at 37 °C for 30 min and visualized with a Leica DMI4000 inverted microscope. Annexin V-positive cells and total cell number were determined using ImageJ software and the percentage of positive cells/total cell number was calculated.

### Generation of HEK-293 cells overexpressing K_Ca_3.1-myc or BirA-myc

300,000 WT HEK-293 cells were seeded in 6-w plates in standard culture medium. The following day, they were transfected with a plasmid codifying K_Ca_3.1-myc (Origene #RC204457) or BirA-myc (pcDNA3.1 mycBioID was a gift from Kyle Roux (Addgene plasmid # 35700; http://n2t.net/addgene:35700)). The transfection was performed in Opti-MEM medium (Gibco) using the LT-1 transfection agent (Mirus). Cells were passed in T-25 flasks and transfected cells were selected in culture medium supplemented with 1 mg/mL Geneticin (Gibco). Resistant cells were maintained in geneticin-containing medium and characterized by Western Blot as described above, using α-myc tag (1:3000 in TTBS, Abcam #ab9106) as primary antibody.

### Wound scratch assay

35,000 B16F10 cells were seeded in 24-well plates in standard culture medium and allowed to grow to confluency for 48 h. The scratch was performed with a sterile p200 pipette tip. The medium was removed and substituted, after one wash in PBS, with phenol red- and serum-free medium, containing the treatment compound at the indicated concentration. For rescue experiments, cells were pre-treated for 1 h with the NF-κB inhibitor TPCA-1 (0.5 μM, Cayman #15115) or the Rho/Rac/Cdc42 Activator I (2.5 μg/ml, Cytoskeleton, Inc. #CN-04A) before performing the scratch. The inhibitors were present throughout the assay. BNIP-3 was overexpressed 24 h after seeding using the LT-1 transfection reagent (Mirus) (pCI BNIP-3 plasmid was a kind gift from Dr. Vanina Romanello [43]). BNIP-3 overexpression was confirmed using Western Blot as described above. Images of the same fields were taken 0, 15, 20, and 24 h after performing the scratch with a Leica DMI4000 inverted microscope. The area of the scratch was calculated using ImageJ software. The migration rate is expressed as % of the initial gap area. For MDA-MB-231 cells, 40,000 cells were seeded in 24-well plates. Cells were serum-starved for 8 h prior to performing the scratch, after which the medium was substituted with phenol-red free medium containing 2% FBS. Treatment, transfection, and co-treatments were performed as described above.

### Soft agar assay

12-well plates were coated with 600 µl phenol red- and serum-free DMEM containing 1% low-melting point agarose (Gibco). Upon solidification, 5000 B16F10 cells/well were seeded in 400 µl phenol red-free DMEM containing 0.6% agarose and 4% FBS. After gelidification 500 µl phenol red-free DMEM containing 1% FBS were added on top of the agarose layers. Two days after seeding, the treatment was started. The compounds were added in the upper layer containing 1% FBS and the medium was replaced every other day. After three weeks, the upper layer was substituted with a solution containing 0.5% (w/v) crystal violet (Sigma-Aldrich) in 20% methanol diluted 1:100 in PBS and incubated for 2 h at RT. The staining solution was removed, and the agarose layers were washed twice with PBS before imaging with a Leica MZ16F stereomicroscope. The area of the colonies was calculated with ImageJ.

### ATP measurements

The amount of ATP was measured in B16F10 cells using the ATPlite Luminescence ATP Detection Assay System (Perkin Elmer). 7000 cells/well were seeded onto white 96-well viewplates. To measure mitochondrial ATP production, cells were maintained and assayed in medium in which glucose had been substituted with 5.5 mM galactose. For total ATP content, treatments were performed in medium containing 5.5 mM glucose. 1 μg/mL oligomycin was used as negative control. 2 h or 24 h after treatment, the assay was performed following the manufacturer’s instructions.

### Quantitative real-time PCR

Cells were seeded in 6-well plates and either left untreated (COLO-357, HPDE, PANC-1, MIA PaCa-2 for K_Ca_3.1 expression) or treated for 24 h with 0.5 µM mitoTRAM-34, 5 µM *rev*-mitoTRAM or the corresponding amount of DMSO (0.1%). Cells were washed in ice-cold PBS and RNA extraction was performed with TRIzol (Thermo Fisher Scientific) following the manufacturer’s protocol. Total RNA was quantified with a NanoDrop 2000 Spectrophotometer (Thermo Fisher Scientific). The SuperScript IV Reverse Transcriptase kit (Thermo Fisher Scientific) was used for retrotranscription of 1 µg RNA as indicated by the supplier using an Eppendorf Mastercycler. The synthetized cDNA was diluted 1:20 and subjected to qRT-PCR with the Power SYBR Green PCR Master Mix (Thermo Fisher Scientific) following manufacturer’s instructions using the CFX384 Touch Real-Time PCR Detection System (BioRad). Tata-box Binding Protein (TBP), Hypoxanthine Phosphoribosyltransferase 1 (HPRT-1) and β-actin were used for normalization. CT values were first normalized with respect to the housekeeping genes (ΔCT) and next compared to the control sample (ΔΔCT). This relative normalized expression is indicated in the figures.

Primers for qRT-PCR:

**Table:** 

Target	Sequence (from 5′ to 3′)	Accession #
h*TBP* (for)	GCACAGGAGCCAAGAGTGAA	NM_003194.5
h*TBP* (rev)	TTGTTGGTGGGTGAGCACAA	
h*HPRT1* (for)	CCTGGCGTCGTGATTAGTGAT	NM_000194.2
h*HPRT1* (rev)	AGACGTTCAGTCCTGTCCATAA	
h*KCNN4* (for)	GTGGGCGCTCTACCTGTTC	NM_002250.3
h*KCNN4* (rev)	CATGAACAGCTGGACCTCTTTG	
m*Tbp* (for)	GCAGTGCCCAGCATCACTAT	NM_013684.3
m*Tbp* (rev)	GCCCTGAGCATAAGGTGGAA	
m*Actb* (for)	AGTGTGACGTTGACATCCGT	NM_007393.5
m*Actb* (rev)	TCGTAGGAGCCAGAGCAGTA	
m*Hif1a* (for)	GAAGTGCACCCTAACAAGCC	NM_001313919.1
m*Hif1a* (rev)	GGGTTCACAAATCAGCACCA	
m*Car9* (for)	GCTGTCCCATTTGGAAGAAA	NM_139305.2
m*Car9* (rev)	GGAAGGAAGCCTCAATCGTT	
m*Snai1* (for)	TCCAAACCACTCGGATGTGAAGA	NM_011427.3
m*Snai1* (rev)	TTGGTGCTTGTGGAGCAAGGACAT	
m*Snai2* (for)	CACATTCGAACCCACACATTGCCT	NM_011415.3
m*Snai2* (rev)	TGTGCCCTCAGGTTTGATCTGTCT	
m*Twist1* (for)	CGGGAGTCCGCAGTCTTA	NM_011658.2
m*Twist1* (rev)	TGAATCTTGCTCAGCTTGTC	
m*Cdh1* (for)	ATTGCAAGTTCCTGCCATCCTC	NM_009864.3
m*Cdh1* (rev)	CACATTGTCCCGGGTATCATCA	
m*Cdh2* (for)	TGAAACGGCGGGATAAAGAG	NM_007664.5
m*Cdh2* (rev)	GGCTCCACAGTATCTGGTTG	
m*Ppargc1a* (for)	GGACATGTGCAGCCAAGACTCT	NM_008904.3
m*Ppargc1a* (rev)	CACTTCAATCCACCCAGAAAGCT	
m*Tfam* (for)	CCAAAAAGACCTCGTTCAGC	NM_009360.4
m*Tfam* (rev)	ATGTCTCCGGATCGTTTCAC	
m*Atp51a* (for)	GAGACTGGGCGTGTGTTAG	NM_007505.2
m*Atp5a1* (rev)	CTCGACGCAATACCATCACCA	
m*Bnip-3* (for)	TTCCACTAGCACCTTCTGATGA	NM_009760.4
m*Bnip-3* (rev)	GAACACCGCATTTACAGAACAA	

### In vivo experiments

WT, 6–12 weeks old male C57BL/6J mice from Envigo were used for all tumor experiments. The number of animals to be used has been calculated using GPower with a pre-specified effect size of 0.54. For injection of melanoma cells subcutaneously in the flank or in the footpad of mice, confluent B16F10 cells were washed in PBS, detached with a trypsin-0.25% EDTA solution (Gibco), counted and resuspended in PBS in an appropriate volume to obtain 50,000 cells/50 µl (subcutaneous injection) or 150,000 cells/50 µl (footpad injection). Mice were anesthetized with alfaxalone 40 mg/kg + xylazine 10 mg/kg before the injection. For orthotopic injection in the pancreas, mice were shaved on the left flank. The surgery site was sterilized with 70% ethanol and betadine. A small incision was performed at the lowest point of the rib cage and the pancreas was exposed. 10^6^ PAN-02 cells were slowly injected in a total volume of 50 µl in a 1:1 solution of PBS and Matrigel (high concentration, Corning #354248). The absence of leakage was controlled at the injection site with a cotton bud. The pancreas was gently slipped back in the peritoneum and the wound was sutured.

All treatments were started 6 days after injection. Mice were randomly divided into two groups and injected every day intraperitoneally with 3 nmol/gbw mitoTRAM-34 (or the corresponding amount of DMSO, max 5 µl in 100 µl saline) in saline solution for 10 days and sacrificed the day after the last injection. The body weight was monitored throughout the treatment. Tumors and organs were harvested after sacrifice and fixed in 3.8% PFA in PBS for 48 h. They were then dehydrated, included in paraffin and cut into 5 µm sections, or immerged in a 30% sucrose solution until the tissue sank, and frozen in OCT. For H&E staining, sections were dewaxed, rehydrated and stained for 3 min with Harris’ modified haematoxylin (Fisher Chemicals) and 30 s of eosin (Sigma-Aldrich). After dehydration, they were mounted with Permount™ mounting medium (Fisher Chemicals) prior to imaging with a Zeiss Axio Observer widefield microscope. For staining of lymph nodes, 10 µm cryosections were thawed for 30 min at room temperature, rehydrated for 30 min in PBS, permeabilized for 10 min in 0.1% Triton X-100 (Sigma-Aldrich) in PBS, washed 3x in PBS, blocked for 1 h in 1% BSA + 5% goat serum in PBS and incubated overnight at 4 °C in 1% BSA + 5% goat serum in PBS with α-Melan-A (1:300, Abcam #ab210546). The next day, sections were washed 3x in PBS and incubated for 1 h at room temperature with secondary antibody anti-rabbit AF488, ThermoFisher #A-11008, 1:1000 in 5% goat serum in PBS). Nuclei were stained with 5 µg/mL Hoechst 33342 (Invitrogen) in PBS for 5 min. Samples were washed 3x, mounted and images were acquired with a Leica DMI4000 inverted microscope. The Melan-A-positive area on the area of the whole lymph node was quantified using ImageJ.

Experiments on mice were conducted with the consent of the Local Ethical Committee at the University of Padua and National Agency, and with the supervision of the Central Veterinary Service of the University of Padova (in compliance with Italian Law DL 116/92 and further modifications, embodying UE directive 86/609), authorization n. 846/2021-PR.

### Statistical analysis

All statistical analyses were performed with GraphPad Prism 9 software. Three or more groups were analyzed with One-Way Anova and Dunnett’s posttest. Two-Way Anova with Bonferroni’s posttest was used to compare data with two variables. Unpaired T tests (between groups with similar variance) and One Sample T tests (comparing an unknown mean to a specific value) were used to compare two groups. Additional statistical details, including exact sample size and replicates, can be found in the figure legends.

## Results

### Synthesis and channel-modulating effects of novel, mitochondria-targeted K_Ca_3.1 inhibitors

**Fig. 1:**
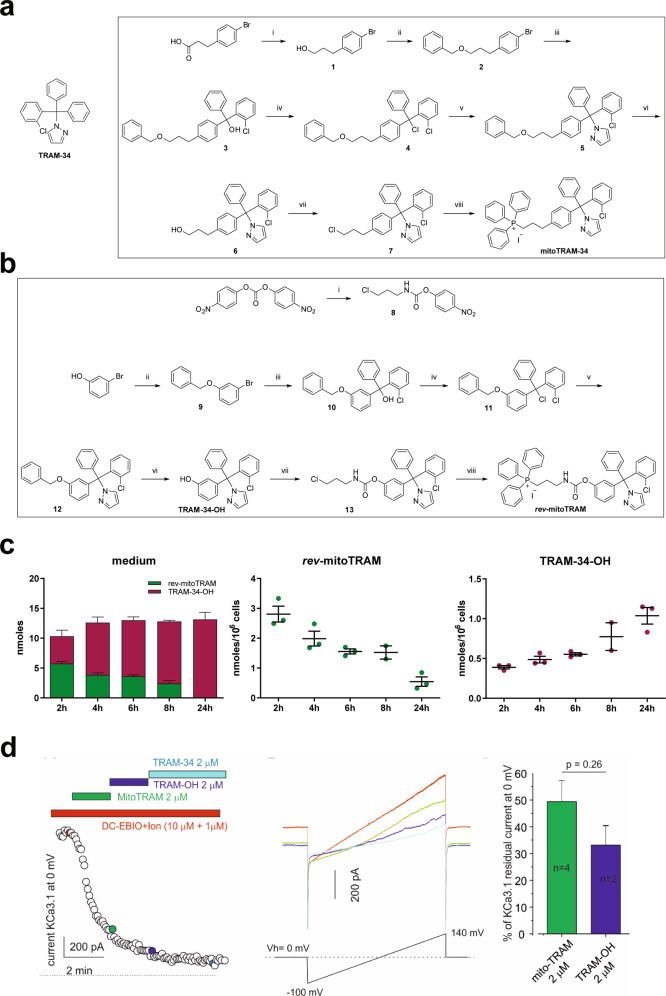
Synthesis and characterization of the novel TRAM-34 derivatives mitoTRAM-34 and *rev*-mitoTRAM. **a** Structure of TRAM-34 (left) and synthesis of mitoTRAM-34 (right). Reagents and conditions: (i) LiAlH_4_, THF, Et_2_O, room temperature for 3.5 h, 94% yield; (ii) (1) NaH, DMF, 0 °C for 1 h; (2) benzyl bromide, room temperature for 3.5 h, 94% yield; (iii) (1) n-butyllithium, THF, −70 °C for 1 h; (2) 2-chlorobenzophenone, from −70 °C to room temperature overnight, 80% yield; (iv) acetyl chloride, toluene, reflux for 45 min; (v) pyrazole, CH_3_CN, reflux overnight, 83% yield; (vi) Pd/C, H_2_, EtOAc, room temperature for 75 min, 90% yield; (vii) LiCl, 2,4,6-trimethylpyridine, methanesulfonyl chloride, DMF, room temperature for 15 h, 89% yield; (viii) NaI, PPh_3_, CH_3_CN, 95 °C for 6 h, 81% yield. **b** Synthesis of *rev*-mitoTRAM. Reagents and conditions: (i) DMAP, 3-chloropropylamine hydrochloride, DCM, THF, room temperature for 3.5 h, 88% yield; (ii) K_2_CO_3_, benzyl bromide, acetone, 70 °C for 15 h, 92% yield; (iii) (1) n-butyllithium, THF, −70 °C for 1 h; (2) 2-chlorobenzophenone, from −70 °C to room temperature overnight, 50% yield; (iv) acetyl chloride, toluene, 100 °C for 1.5 h; (v) pyrazole, CH_3_CN, 100 °C for 17 h, 68% yield; (vi) Pd/C, H_2_, EtOAc, room temperature for 1.5 h, 87% yield; (vii) DMAP, 8, CH_3_CN, 50 °C for 20 h, 82% yield; (viii) NaI, PPh_3_, CH_3_CN, 95 °C for 6 h, 72% yield. **c** Levels of *rev*-mitoTRAM and its hydrolysis product (TRAM-34-OH) in the medium and in B16F10 cells after 2, 4, 6, 8, or 24 h of incubation with 5 µM *rev*-mitoTRAM. Mean ± SEM, *N* = 3. **d** MitoTRAM-34 and TRAM-34-OH block plasma membrane K_Ca_3.1 currents in GL-15 cells. Left: Time-course of the current at 0 mV measured from the current ramps obtained by applying linear gradients of potential from −100 to 140 mV (Vh of 0 mV) repeated every 5 s. 0 pA current level is indicated by the dashed line. The K_Ca_3.1 current was activated by DC-EBIO (1 mM) and ionomycin (500 nM) (EBIO/ionomycin), and the chamber was then perfused sequentially (in the presence of the activators) with mitoTRAM-34, TRAM-34-OH and finally with TRAM-34 (all 2 µM). Individual data points in the trace represent the current conducted by K_Ca_3.1 channels following activation by EBIO/ionomycin and inhibition by the indicated drugs. Center: Representative current ramps recorded at the time points indicated on the left panel (same color code). Right: Mean residual K_Ca_3.1 current recorded at 0 mV after mitoTRAM-34 and TRAM-34-OH application, expressed as % of the difference between the current recorded after full activation and after complete block of K_Ca_3.1 with 3 µM TRAM-34 (*n* = 5). Leak current was not subtracted.

Here we describe the development and characterization of two mitochondriotropic TRAM-34 derivatives, named mitoTRAM-34 and *rev*-mitoTRAM. First, we used a short, chemically stable alkyl chain (C3) to link a positively charged, lipophilic triphenylphosphonium (TPP^+^) moiety [44] to TRAM-34 (mitoTRAM-34) in a rational synthesis approach based on previously published structure-activity relationship studies (Fig. 1a) [27, 45]. The TPP^+^ moiety drives a preferential accumulation of the drugs linked to it into the mitochondria, given the highly negative mitochondrial membrane potential (Δψ_m_) (around –180 mV) [46]. TRAM-34 is highly lipophilic, and thus able to cross biological membranes, and exhibits high potency and selectivity for K_Ca_3.1 [25]. We further synthetized the prodrug *rev*-mitoTRAM, in which the TPP^+^ group has been linked to TRAM-34 by a carbamate ester bond that can be processed by cellular esterases, resulting in the release of TPP-propyl-NH_3_ (TPPP-NH_3_) and TRAM-34-OH, expectedly after accumulation of the drug in the polarized mitochondria (Fig. 1b). TRAM-34-OH differs from TRAM-34 only for the presence of an hydroxyl group on one of the phenyl rings (Fig. 1a, b). In incubations with intact cells, *rev*-mitoTRAM was able to release TRAM-34-OH in a time-dependent manner, as expected: after 2 h of treatment of B16F10 mouse melanoma cells with 5 µM *rev*-mitoTRAM, a sub-lethal concentration, about one-half of the prodrug had been hydrolyzed to TRAM-34-OH. After 24 h, only TRAM-34-OH could be detected in the medium (Fig. 1c, left panel). Accordingly, cell pellets progressively accumulated TRAM-34-OH while the amount of *rev*-mitoTRAM decreased over time (Fig. 1c, center and right panels). After 2 h of treatment, we found approximately 0.4 nmol TRAM-34-OH/10^6^ cells, which corresponds, assuming a cell volume of 3000 femtoliters [47], to a concentration of ≈100 µM inside the cells (assuming that no fraction of the molecule is bound to membranes). This extent of enrichment is typical of TPP^+^-linked molecules [48].

Molecular docking analysis, performed following previous studies [27] based on the structure of two K^+^ channels (KcsA and Kv1.2 pore region), showed the predicted positions of TRAM-34, TRAM-34-OH and mitoTRAM-34 in KscA-based (Figure S1a) and Kv1.2-based (Figure S1b) models (for comparison see Figure S1c). The best docking scores were obtained using the K_Ca_3.1 homology model based on KcsA [49] and the best positions, for the two models and all three ligands (TRAM-34, mitoTRAM-34, and TRAM-OH), are shown in Table 1. Our analyses predicted that TRAM-34-OH would indeed be able to enter the internal cavity of K_Ca_3.1 occupying a position similar to the one occupied by TRAM-34. To experimentally verify the K_Ca_3.1 channel-inhibiting properties of the new TRAM-34 derivatives, we performed patch clamp experiments in the well-established GL-15 model [39]. In a whole-cell configuration, following the application of potassium channel openers DC-EBIO and ionomycine (Ion), we observed an increase of outward currents at 0 mV, evaluated by applying voltage ramps from −100 to 140 mV (Vh = 0 mV) at 5-sec intervals (data not shown). The reversal potential of (DC-EBIO + Ion)-triggered currents was close to the equilibrium potential of potassium ions in our ionic conditions, as calculated using Nernst’s equation (Fig. 1d, central panel). The figure illustrates the blocking properties of 2 µM mitoTRAM-34, TRAM-34-OH, and TRAM-34. The compounds were applied in series to better evaluate their effectiveness as blockers (Fig. 1d, left panel). MitoTRAM-34 blocks the K_Ca_3.1 currents with a half-block concentration close to 2 µM. TRAM-34-OH is slightly, but not significantly, more efficient (Fig. 1d, right panel, *p* = 0.26). Although both blockers (mitoTRAM-34 and TRAM-34-OH) are less potent inhibitors of K_Ca_3.1 than TRAM-34 (*p* < 0.05), they can be considered efficacious blockers of endogenous K_Ca_3.1 currents.

**Table 1:** EC_50_ values (μM) of different cancer cell lines of mitoTRAM-34 and *rev*-mitoTRAM.

Cell line	mitoTRAM	*rev*-mitoTRAM
B16F10	1.665 ± 0.2086	7.912 ± 0.5313
MDA-MB-231	1.317 ± 0.07186	4.964 ± 0.2451
COLO-357	1.532 ± 0.2004	9.195 ± 1.075
MiaPACA-2	2.498 ± 0.07353	9.06 ± 1.316
PANC-1	3.03 ± 0.2834	11.61 ± 0.8049
PAN-02	2.255 ± 0.07343	8.768 ± 1.281

Viability was assessed using MTS assays. The concentration at which 50% of the cells were viable was calculated using the curve-fitting function of OriginLab (mean + SEM, *N* = 3–5).

### MitoTRAM-34 and *rev*-mitoTRAM strongly affect mitochondrial physiology

As mentioned above, the TPP^+^ moiety of the novel TRAM-34 derivatives drives a rapid accumulation into mitochondria [44] and is likely to enhance solubility (the solubility of TRAM-34 is rather low [28]). Thus, we evaluated the effects of mitoTRAM-34 and *rev*-mitoTRAM on different mitochondrial parameters. We chose B16F10 cells as a model system for these studies, because (i) a high expression of K_Ca_3.1 can be observed in numerous melanoma cell lines (Cancer Cell Line Encyclopedia, https://portals.broadinstitute.org/ccle/page?gene=KCNN4) as well as in human skin cancer samples (The Human Protein Atlas, https://www.proteinatlas.org/ENSG00000104783-KCNN4/pathology); (ii) TRAM-34 was shown to increase the sensitivity of human melanoma cells to tumor necrosis factor-related apoptosis-inducing ligand (TRAIL) treatment, suggesting an involvement of mitoK_Ca_3.1 [19]; (iii) B16F10 cells are syngeneic to C57BL/6 mice, allowing assessment of effects of drugs on tumor growth in vivo in immune-competent mice.

**Fig. 2:**
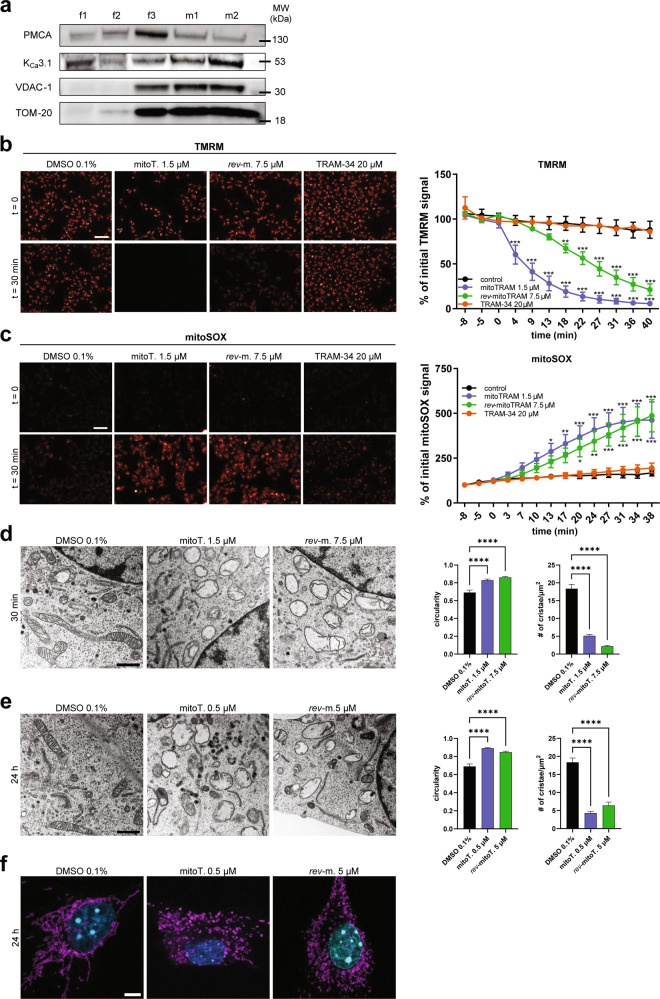
Effects of mitoTRAM-34 and *rev*-mitoTRAM on mitochondrial physiology. **a** Representative Western Blot showing the mitochondrial localization of K_Ca_3.1 in B16F10 cells. f1: whole-cell extract; f2: membrane-enriched fraction; f3: mitochondria-enriched fraction; m1 and m2: Percoll-purified mitochondrial fractions (see “Materials/subjects and methods”). Plasma membrane marker PMCA and mitochondrial membrane markers VDAC-1 and TOM-20 are also shown. **b**, **c** Images showing mitochondrial membrane potential changes (**b**) and superoxide production (**c**) in B16F10 cells, as visualized by changes in TMRM (**b**) or mitoSOX (**c**) fluorescence upon addition of mitoTRAM-34 (mitoT.), *rev*-mitoTRAM (*rev*-m.) or TRAM-34 at the indicated time points. FCCP (**b**) and Antimycin A (**c**) were used as controls (not shown). The quantification of the fluorescence signal is shown on the right. Fluorescence is expressed as percentage of the initial intensity (mean + SEM; Two-Way Anova with Dunnett’s multiple comparison test. *N* = 4. *p < 0.05, **p < 0.01, ***p < 0.001 compared to control). Scale bar is 100 µm. **d** Representative images (left) and analysis (right) of mitochondrial ultrastructure assessed by transmission electron microscopy (TEM) after treatment of B16F10 cells with 1.5 µM mitoTRAM (mitoT.) or 7.5 µM *rev*-mitoTRAM (*rev*-m.) for 30 min. The mitochondrial circularity and number of cristae/area were calculated with ImageJ. For each replicate, mitochondria from at least 5 cells were analyzed (mean + SEM, One-Way Anova with Dunnett’s posttest. *N* = 3. ****p < 0.0001 compared to control). Scale bar is 1 µm. **e** As in (**d**), but cells were treated for 24 h with sublethal doses of mitoTRAM-34 (0.5 µM) and *rev*-mitoTRAM (5 µM). **f** Representative confocal images of B16F10 cells treated as in (**e**) showing a fragmented mitochondrial network after 24 h of treatment with mitoTRAM-34 (mitoT.) or *rev*-mitoTRAM (*rev*-m.) compared to control cells. Cells were stained for mitochondrial marker TOM-20 (magenta) and DAPI (cyan). Scale bar is 5 µm.

We first verified the expression of mitoK_Ca_3.1 in B16F10 melanoma cells. As shown in Fig. 2a, we observed a progressive enrichment of the mitochondrial marker proteins voltage-gated anion channel 1 (VDAC-1) and TOM-20 in the purified (m1) and highly purified (m2) mitochondrial fractions when compared to whole-cell lysates (f1 and f2) and to the membrane-enriched fraction (f3). In parallel with mitochondrial enrichment, the relative amount of PM in the different fractions decreased, as shown by the levels of the PM calcium ATPase (PMCA), while K_Ca_3.1 levels increased (Fig. 2a). These data strongly indicate the presence of this channel in B16F10 mitochondria.

Mitochondrial K^+^ channels regulate matrix volume, respiration, membrane potential, and ROS generation [1]. Thus, we tested the effects of TRAM-34 and its mitochondriotropic derivatives on mitochondrial ROS production and membrane potential. The EC_50_ values calculated from cell viability assays (see below) were used in these assays: 1.5 µM for mitoTRAM-34 and 7.5 µM for *rev*-mitoTRAM-34. As previously reported for other mitochondrial potassium channels [7, 8], inhibition of mitoK_Ca_3.1 by mitoTRAM-34 and *rev*-mitoTRAM led to the loss of the inner mitochondrial membrane potential (Δψ_m_) (as visualized by the decrease in TMRM fluorescence, Fig. 2b) and to a significant increase in mitochondrial superoxide production (as indicated by the increase in mitoSOX fluorescence, Fig. 2c). Increased ROS production and loss of Δψ_m_ were not a consequence of the accumulation of TPPP-NH_3_ in mitochondria, as this compound itself had no effect either on mitoSOX or on TMRM fluorescence (Fig. S2a). Similarly, neither TRAM-34 nor the membrane-impermeant K_Ca_3.1 inhibitor maurotoxin (used at 200 nM, in excess with respect to the IC_50_ value of 1.1 nM [50]), which acts exclusively on PM K_Ca_3.1, affected these parameters (Fig. 2b, c and Fig. S2a). The lack of effect of TRAM-34 is most likely due to its lipophilic nature and low solubility. In support of this hypothesis, the more soluble TRAM-34-OH (the hydrolysis product of *rev*-mitoTRAM), used as such, induces a moderate increase in ROS production, which, however, is not sufficient to cause depolarization of the inner mitochondrial membrane (Fig. S2a).

**Fig. 3:**
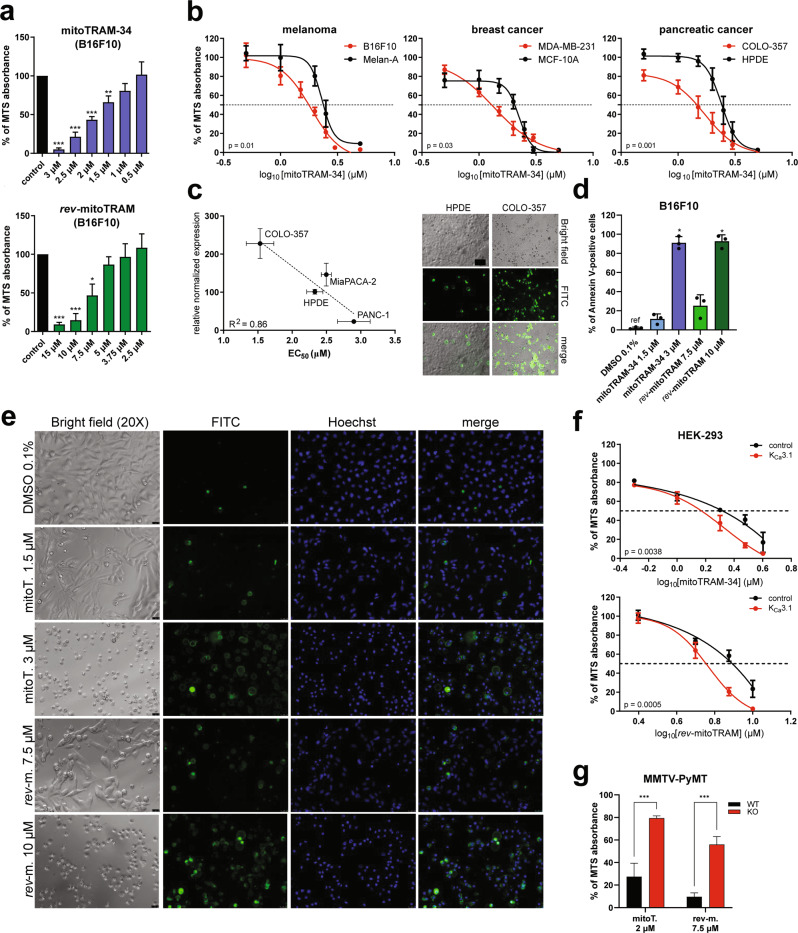
The novel TRAM-34 derivatives dose-dependently induce cell death in a variety of cancer cells. **a** MTS assays showing the sensitivity of B16F10 cells to different doses of mitoTRAM-34 and *rev*-mitoTRAM (24 h). Shown are means + SEM of *N* = 3–5 (One Sample T Test, ***p-value < 0.001, **p-value < 0.01, *p-value < 0.05). **b** Dose-response curves showing the sensitivity of the cancer cell lines B16F10, MDA-MB-231, and COLO-357 and non-tumoral cell lines from the same tissues (Melan-A, MCF-10A, and HPDE, respectively) to mitoTRAM-34, as assessed by MTS assays. The concentrations applied (µM) are plotted on a logarithmic scale. The concentration at which 50% of cells were viable (EC_50_) is indicated by the dotted line. Shown are means + SEM and nonlinear fit curves of *N* = 3–5. Statistical differences between curves were analyzed with Two-Way Anova. **c** EC_50_ values for mitoTRAM-34 treatment of different human pancreatic cancer cell lines and a non-tumoral pancreatic epithelial cell line (HPDE) of mitoTRAM-34 treatment assessed with MTS assays plotted against the expression of K_Ca_3.1 in the same cells, analyzed with qRT-PCR. The correlation was analyzed with a simple linear regression. Plot of mean ± SEM EC_50_ values vs. normalized expression relative to HPDE cells (*N* = 3). On the right, representative images of Annexin V-FITC stainings of HPDE and COLO-357 cells treated for 24 h with 1.5 µM mitoTRAM-34. Bright field images, FITC fluorescence and merge are shown. Scale bar is 100 µm. **d** Quantification of Annexin V-FITC staining shown in (**e**). The fraction of Annexin V-positive cells on total cell count is shown (mean + SEM, One-Way Anova with Dunnett’s posttest. *N* = 3. *p < 0.05). **e** Representative images of an Annexin V-FITC cell death assay. B16F10 cells were treated for 24 h with mitoTRAM-34 (mitoT.) or *rev*-mitoTRAM (*rev*-m.) at the indicated concentrations were stained with Annexin V-FITC. Bright field images, FITC fluorescence, Hoechst staining and the merged images are shown. Scale bar is 25 µm. **f** Sensitivity of HEK-293 cells overexpressing either K_Ca_3.1 or the control protein BirA to mitoTRAM-34 or *rev*-mitoTRAM treatment analyzed with MTS assays. The concentration at which 50% of cells were viable (EC_50_) is indicated by the dotted line. Shown are mean + SEM and nonlinear fit curves of *N* = 4. Statistical differences between curves were analyzed using Two-Way Anova. **g** Cell viability of WT and K_Ca_3.1 KO primary breast cancer cells after 24 h of treatment with 2 µM mitoTRAM-34 (mitoT.) or 7.5 µM *rev*-mitoTRAM (*rev*-m.), analyzed by MTS assays. For both WT and KO cells, data from 2 separate primary cultures were put together (mean + SEM, Two-Way Anova with Sidak’s multiple comparisons tests. *N* = 3. ***p < 0.001).

MitoTRAM-34 and *rev*-mitoTRAM also rapidly and strongly affected mitochondrial ultrastructure. When applied at the same concentrations used above (EC_50_ in viability tests), they led to mitochondrial swelling and fragmentation as well as loss of cristae structure within 30 min (Fig. 2d). Long-term treatment (24 h) of B16F10 cells with sub-lethal concentrations (0.5 µM mitoTRAM-34 and 5 µM *rev*-mitoTRAM) induced loss of ultrastructure and mitochondrial fragmentation as shown in Fig. 2e, f, while cells were still 100% viable (Fig. 3a).

Despite the drastic and rapid ultrastructural changes observed at higher concentrations of the drugs, neither one had a drastic effect on stimulated and maximal respiration. Instead, they dose-dependently increased oligomycin-resistant respiration in Seahorse experiments (Fig. S2b, c). Since we also observed a decreased ATP-linked respiration (Fig. S2b, c), we analyzed complex V activity in isolated B16F10 mitochondria. As a readout we used a spectrophotometric assay which follows the activity of ATP synthase (complex V) in the reverse mode, i.e., the hydrolysis of ATP (see “Materials/subjects and methods” section). Surprisingly, TRAM-34 and its novel derivatives dose-dependently reduced the hydrolytic activity of ATP synthase in B16F10 and mouse liver mitochondria (Fig. S2d, e) without, however, affecting ATP synthesis (Fig. S2f), the activity of enzymes employed in the assay (Fig. S2g), or that of complexes I and III (Fig. S2h, i).

### MitoTRAM-34 and *rev*-mitoTRAM dose-dependently induce cell death in a variety of cancer cell lines

We then analyzed the effects of the novel TRAM-34 derivatives on cell viability, using the MTS assay. Both mitoTRAM-34 and *rev*-mitoTRAM dose-dependently reduced the viability of B16F10 melanoma cells, although at different concentrations (Fig. 3a). In addition to B16F10 cells, mitoTRAM-34 efficiently decreased cell viability in the triple-negative breast cancer line MDA-MB-231 and in different pancreatic cancer cell lines. EC_50_ values ranged from 1.3 to 3 µM (Fig. 3b and Table 1). Notably, cancer cells were more sensitive to mitoTRAM-34 than corresponding non-tumoral cell lines from the same tissues (Fig. 3b). In the tested subset of different pancreatic cell lines, which have been previously reported to express functional K_Ca_3.1 in mitochondria [18], channel expression analyzed by qRT-PCR correlated with the sensitivity of the cell lines to mitoTRAM-34, suggesting specificity of the drug action through K_Ca_3.1 (Fig. 3c). *Rev*-mitoTRAM also dose-dependently reduced cell viability in the tested cell lines, although at higher EC_50_ values. The difference can be attributed to the hydrolysis taking place in intact cells and the subsequent free diffusion of TRAM-34-OH to other cellular compartments or outside the cell (Table 1). In agreement with this notion, TRAM-34-OH (which is not selectively accumulated in mitochondria but freely diffuses) affected cell viability at much higher concentrations than *rev*-mitoTRAM (Fig. S3a). Neither TRAM-34 nor TPPP-NH_3_ had any effect on cellular survival at the highest used dose (20 µM) (Fig. S3b). The lack of effect on cell viability by TRAM-34 is in agreement with previous studies (e.g., [24]). To confirm that the reduced MTS absorbance corresponds to cell death induction and not just to a metabolic quiescent state, we stained treated cells with Annexin V-FITC and Hoechst dyes (Figs. 3c, d and S3c). Both mitoTRAM-34 and *rev*-mitoTRAM dose-dependently increased the number of FITC-labeled Annexin V-positive cells and induced death in nearly all cells when applied for 24 h at 3 µM or 10 µM, respectively (Fig. 3e). Under these conditions, the nuclei appeared condensed, further suggesting that apoptosis has occurred [51]. Since ROS production and Δψ_m_ loss were dose-dependent (Fig. S3e), we suggest that full depolarization occurs above a certain ROS level and leads to cell death. Reflecting the MTS results, dead cells were not detected in TPPP-NH_3_-treated samples; TRAM-34 induced cell death in ≈10% of cells, while TRAM-34-OH was more effective, killing 40% of B16F10 cells at the same concentration (20 µM) (Fig. S3c, d).

Overexpression of K_Ca_3.1 in HEK-293 cells, which normally do not express K_Ca_3.1 [52], significantly increased their sensitivity to mitoTRAM-34 and *rev*-mitoTRAM (Fig. 3f). Viceversa, MMTV-PyMT primary breast cancer cells from WT mice were much more sensitive to the drugs than the cancer cells isolated from K_Ca_3.1-KO mice [20] (Fig. 3g), indicating again that cell death-inducing effects indeed prevalently depend on mitoK_Ca_3.1 expression. To check a possible effect of the ATP-ase inhibiting action of the drugs on cell survival, the sensitivity of B16F10 to the novel TRAM-34 derivatives was tested under hypoxic (1% O_2_) conditions. A reduced oxygen tension can induce cells to switch from an aerobic to an anaerobic mode to sustain Δψ_m_ and under these conditions inhibition of ATP hydrolysis by complex V occurs [53, 54]. We found no significant difference in cell survival upon treatment under normoxic or hypoxic conditions (Fig. S3f). An inhibitory effect of mitoTRAM-34 on ATP hydrolysis is thus unlikely to contribute significantly to cell death.

### At sub-lethal concentrations, mitoTRAM-34 and *rev*-mitoTRAM reduce cellular migration and resistance to *anoikis*

**Fig. 4:**
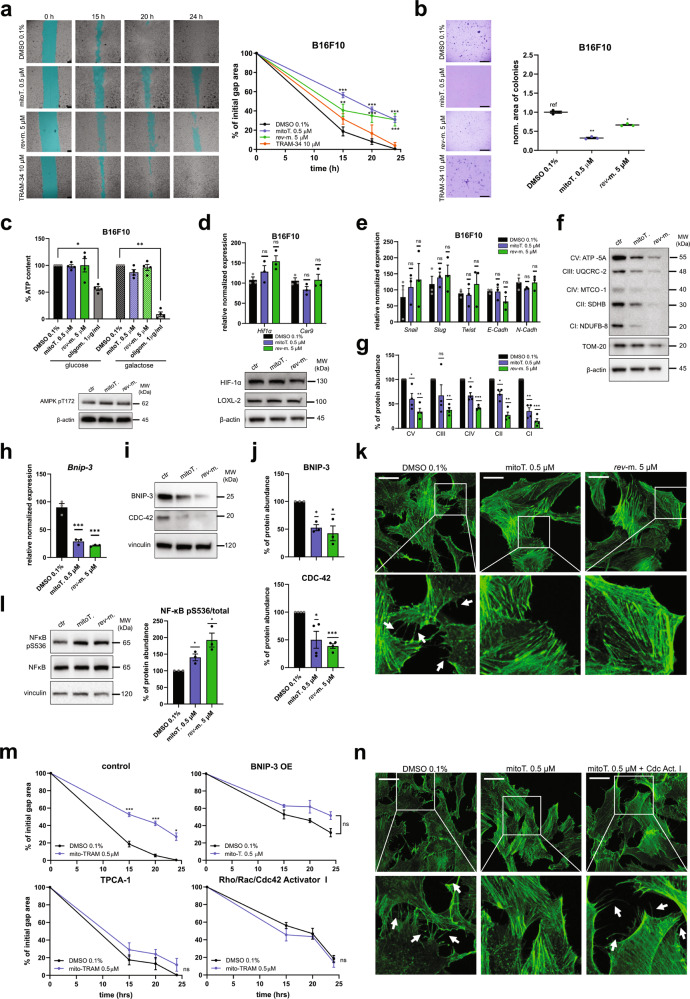
Sublethal doses of mitoTRAM-34 and *rev*-mitoTRAM reduce migration and *anoikis* resistance of B16F10 cells by affecting the cytoskeletal architecture. **a** Representative images (left) and quantification (right) of wound scratch assays in B16F10 cells. Cells were treated with 0.5 µM mitoTRAM-34 (mitoT.), 5 µM *rev*-mitoTRAM (*rev*-m.), or 10 µM TRAM-34. The area of the gap has been highlighted in cyan for better visualization. The gap area was measured 0, 15, 20, and 24 h after performing the scratch using ImageJ and expressed as % of the initial gap area (mean + SEM, Two-Way Anova with Dunn’s posttest. *N* = 3–4. **p-value < 0.01, ***p-value < 0.001 compared to control). Scale bar is 250 µm. **b** Representative images showing the colony growth of B16F10 cells treated as in (**a**) in soft agar assays. The quantification on the right shows the total area of colonies measured with ImageJ and normalized to the control. However, small precipitates in TRAM-34-treated samples (likely due to the known low solubility of TRAM-34 [28]) were observed in soft agar assays therefore quantification of these samples are not reported in the graph. Mean + SEM of *N* = 3 is shown. Data were analyzed with One-Way Anova with Dunnett’s posttest (*p < 0.05, **p < 0.01 compared to the control). Scale bar is 5 mm. **c** The amount of ATP compared to the control in B16F10 cells treated for 24 h as indicated. 1 µg/ml oligomycin was used as negative control. Cells were cultured either in glucose or in galactose (that maximizes oxidative phosphorylation) to distinguish between total cellular and mitochondrial ATP production. Mean + SEM of *N* = 4 are shown (one-sample T test. *p < 0.05, **p < 0.01). Below, a representative Western Blot showing phosphorylated AMPK. β-actin was used as loading control. The quantification is shown in Fig. S4b. **d** Upper panel: Expression of HIF-1α and its target gene CA-IX in B16F10 cells after treatment for 24 h as indicated, analyzed by qRT-PCR. The normalized expression level relative to the control is reported (mean + SEM, *N* = 3). One-Way Anova with Dunnett’s posttest. ns: not significant). Below, representative Western Blot showing protein levels of HIF-1α and LOXL-2. β-actin was used as loading control. Quantification is shown in Fig. S4c. **e** Expression of genes related to epithelial-to-mesenchymal transition in B16F10 cells treated for 24 h as indicated. Data were analyzed as in (**d**). **f** Representative Western Blot of B16F10 cells treated for 24 h with 0.5 µM mitoTRAM-34 (mitoT.) or 5 µM *rev*-mitoTRAM (*rev*-m.) showing oxidative phosphorylation complexes I-V (CI-CV), mitochondrial outer membrane protein TOM-20 and β-actin (loading control). Quantification is shown in (**g**). **g** Quantification of the Western Blot shown in (**f**). TOM-20 was used for normalization and protein expression in treated samples is expressed as % compared to control (mean + SEM, *N* = 4. One-sample T test. ns: not significant, *p < 0.05, **p < 0.01, ***p < 0.001). **h** Expression of BNIP-3 in B16F10 cells after treatment for 24 h as indicated, analyzed by qRT-PCR. The normalized expression level relative to the control is reported (mean + SEM, *N* = 3. One-Way Anova with Dunnett’s posttest. ***p < 0.001). **i** Representative Western Blots showing protein levels of BNIP-3 and CDC-42 treated as in (**h**). Quantification is shown in (**j**). **j** Quantification of the Western Blots shown in (**i**). Vinculin was used for normalization and protein expression in treated samples is expressed as % compared to control (mean + SEM, *N* = 3–4. One-sample T test. *p < 0.05, ***p < 0.001). **k** Confocal images of phalloidin staining in B16F10 cells treated for 24 h with 0.5 µM mitoTRAM-34 (mitoT.) or 5 µM *rev*-mitoTRAM (*rev*-m.). Multiple filopodial extensions are visible in control cells (indicated by white arrows) that are mostly lacking in treated samples. Scale bar is 15 µm. **l** Representative Western Blot showing protein levels of phosphorylated and total NF-κB in B16F10 cells treated as indicated for 24 h. Quantification is shown on the right panel. Vinculin was used for normalization and protein expression in treated samples is expressed as % compared to control (mean + SEM, *N* = 3. One-sample T test. *p < 0.05). **m** Quantification of wound scratch assays in B16F10 cells. Cells were treated with 0.5 µM mitoTRAM-34 (mitoT.) alone or together with NF-κB inhibitor TPCA-1 (0.5 µM), Rho/Rac/Cdc42 Activator I (2.5 µg/ml) or BNIP-3 overexpression (OE). The gap area was measured 0, 15, 20, and 24 h after performing the scratch using ImageJ and expressed as % of the initial gap area (mean + SEM, Two-Way Anova with Dunn’s posttest. *N* = 3–4. *p-value < 0.05, ***p-value < 0.001 compared to control). Representative images are shown in Fig. S4h. **n** As in (**k**), but cells were treated either with 0.5 µM mitoTRAM-34 (mitoT.) alone or together with 2.5 µg/ml Rho/Rac/Cdc42 Activator I (Cdc Act. I), which largely restores filopodal extensions (white arrows). Scale bar is 15 µm.

Given the involvement of K_Ca_3.1 in cancer cell migration and metastasis formation [55], we wondered whether mitoK_Ca_3.1 might play a role in these fundamental processes. Both mitoTRAM-34 and *rev*-mitoTRAM, when applied at doses (0.5 µM and 5 µM) that do not affect cell survival (Fig. 3a, b), significantly decreased the migratory ability of B16F10 cells in wound healing assays (Fig. 4a and Fig. S4a). Resistance to *anoikis*, an important step in the promotion of invasion [56], was also drastically reduced, as assessed by soft agar assays (Fig. 4b). TRAM-34 itself did not affect migration (Fig. 4a) or colony formation (Fig. 4b).

We next sought to determine the mechanism by which the mitochondria-targeted K_Ca_3.1 inhibitors slow down migration and anchorage-independent growth. These phenomena depend on ATP levels, 5’ AMP-activated protein kinase (AMPK) signaling, pathways mediated by ROS and hypoxia-inducible factor (HIF)-1α, epithelial-to-mesenchymal transition (EMT) and Rho-GTPase-dependent signaling [56–59]. First, we analyzed ATP levels in cells treated with the above-mentioned sub-lethal doses of the drugs. Surprisingly, at these low concentrations no significant differences in the amount of cellular ATP were detected upon treatment for 2 (Fig. S4b) or 24 h (Fig. 4c), not even when the cells were cultured in galactose rather than in glucose, a condition that enhances mitochondrial oxidative phosphorylation at the expense of glycolysis (Fig. 4c, upper panel). This result was coherent with a lack of activation of AMPK, revealed by the analysis of phosphorylation at Thr172 (Fig. 4c, lower panel and Fig. S4c). These observations suggest that, per se, an acute modulation of mitoK_Ca_3.1 activity in B16F10 melanoma cells does not alter the cellular energetic state. Next, we checked the expression of HIF-1α at both mRNA and protein levels. HIF-1α expression did not significantly increase in mitoTRAM-34- and *rev*-mitoTRAM-treated cells, and accordingly, no differences were detected in the expression of carbonic anhydrase IX (CA-IX, Car9) and Lysyl Oxidase-Like 2 (LOXL-2) (Figs. 4d and S4d). Both these latter proteins are regulated by HIF-1α-dependent transcription and are involved in the promotion of the invasive phenotype [60–62]. Similarly, we found no significant variations in the expression of genes related to the EMT, such as cadherins, *Twist, Snail*, and *Slug* (Fig. 4e).

Given the strong effects on mitochondrial cristae structure and network organization (Fig. 2e, f), we analyzed the abundance of proteins belonging to respiratory chain complexes I-V. Both mitoTRAM-34 and *rev*-mitoTRAM significantly reduced the amount of the protein markers of all five complexes embedded in the IMM, which is in line with the loss of cristae organization (Fig. 4f, g) [63]. The expression of master regulators of mitochondrial transcription and biogenesis (Transcription Factor A, Mitochondrial (TFAM) and Peroxisome proliferator-activated receptor Gamma Coactivator (PGC)-1α, respectively) or complex V subunit ATP-5A was unaffected at the transcriptional level, suggesting an effect at the translational level (Fig. S4e). Importantly, the protein abundance of OMM protein TOM-20 did not change, indicating a specific effect on inner mitochondrial membrane structure (Figs. 4f and S4f).

Bcl-2 Nineteen kD-Interacting Protein (BNIP-3) affects mitochondrial morphology [64], and K_Ca_3.1 activity has recently been linked to mitochondrial quality control and BNIP-3 expression [65]. Therefore, we next analyzed the mRNA and protein levels of BNIP-3. This BH3-only protein is an important participant in mitochondrial quality control processes and regulates both apoptosis and autophagy [66]. Interestingly, upon treatment we observed a strong downregulation of *Bnip-3* expression levels, which correlated with a 50% decrease in protein abundance (Fig. 4h–j). It has previously been shown in B16F10 cells that BNIP-3 knockdown (i) reduces clonogenic expansion, (ii) increases mitochondrial fission, and (iii) decreases cellular migration by modulating the actin cytoskeleton [67]. Accordingly, in our samples we observed a strongly reduced colony growth in soft agar assays (Fig. 4b), increased mitochondrial fission (Fig. 2f) and reduced migration (Fig. 4a and Fig. S4a) that correlated with low BNIP-3 levels. We therefore analyzed the protein level of CDC-42, a Rho GTPase important for filopodia formation [68], and found a significant reduction (Fig. 4i, j). In agreement with Maes et al. [67], this correlated with a decreased formation of filopodial extensions in B16F10 cells treated with the mitoK_Ca_3.1 inhibitors (Fig. 4k and Fig. S4g). The downregulation of BNIP-3 could be explained by the observed phosphorylation of NF-κB, which has been shown to control BNIP-3 expression [69] and is regulated by ROS [70] (Fig. 4l).

To confirm that mitoK_Ca_3.1 inhibition is linked to reduced migration through a signaling cascade involving (i) NF-κB phosphorylation/activation; (ii) BNIP-3 downregulation; and (iii) CDC-42 reduction, we investigated whether mitoTRAM-34 was still able to reduce the migratory ability of B16F10 cells in which (i) NF-κB was inhibited using TPCA-1 [71]; (ii) BNIP-3 was overexpressed (see Fig. S4i); (iii) CDC-42 was activated using the Rho/Rac/Cdc42 Activator I [72]. Following these interventions, mitoTRAM-34 was unable to reduce migration (Fig. 4m and Fig. S4h) and filopodial extension was restored upon co-treatment with Rho/Rac/Cdc42 Activator I even in the presence of mitoTRAM-34 (Fig. 4n). The same effects could be observed also in MDA-MB-231 cells upon BNIP-3 overexpression (Fig S4i) and activation of Rho/Rac/Cdc42 (Fig. S4j).

### MitoTRAM-34 reduces tumor growth and metastasis to lymph nodes in vivo

**Fig. 5:**
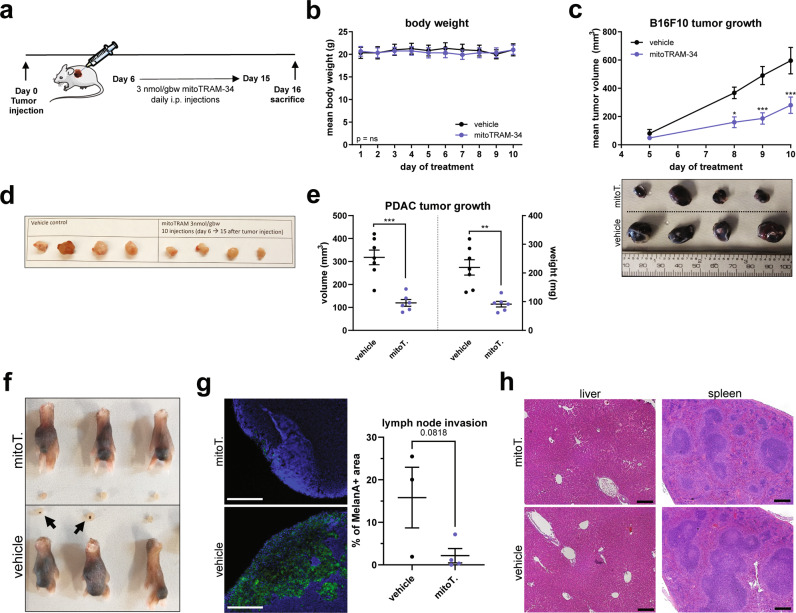
The novel TRAM-34 derivatives reduce tumor growth in vivo in a melanoma and pancreatic cancer model. **a** Treatment scheme for mice. 6 days after tumor injection, mice were treated daily intraperitoneally (i.p.) with 3 nmol/gram body weight (gbw) mitoTRAM-34 for 10 days and sacrificed the day after the last treatment. **b** Body weight of vehicle- vs mitoTRAM-34-treated mice during treatment (mean + SEM, *N* = 4–6. Two-Way Anova. ns = not significant). **c** Tumor volume of B16F10 cells injected subcutaneously, measured at different time points during the treatment with a digital caliper. The volume was calculated using the formula (a*b^2^)/2. *N* = 4 (control) and 6 (mitoTRAM-34). Tumors isolated at the end of the treatment are shown below. Mean + SEM are reported (Two-Way Anova with Bonferroni’s multiple comparison test. *p < 0.05, ***p < 0.001). **d** Representative images of vehicle- or mitoTRAM-34 treated pancreatic tumors (orthotopic injection of PAN-02 cells). **e** Scatter plots showing mean + SEM of pancreatic tumor volume (calculated (a*b^2^)/2) and weight. a and b correspond to the length (**a**) and width (**b**) of the tumor. *N* = 6 (mitoTRAM-34) and 7 (control) (Unpaired T-test. **p < 0.01, ***p < 0.001). **f** For lymph node infiltration, B16F10 cells were injected in the footpad of mice, which were treated as in (**a**). Feet and popliteal lymph nodes of treated vs control mice are shown. No black dots (melanoma cells) are visible in the lymph nodes of mitoTRAM-34-treated mice, while present in the vehicle control (black arrows). *N* = 3 (control) and 4 (mitoTRAM-34). **g** Representative images of lymph nodes shown in (**f**) stained with Melan-A (green, specific for melanoma cells) and Hoechst (nuclei) isolated from mitoTRAM-34 (mitoT.)-treated or control mice. Quantification of the Melan-A-positive area on the total area of the lymph node is shown on the right (mean + SEM, unpaired T-test. *N* = 3–4. *p*-value is indicated). Scale bar is 200 µm. **h** Representative images of H&E stainings of liver and spleen sections of vehicle- and mitoTRAM-34-treated mice as indicated. The treatment regimen is shown in (**a**). Scale bar is 250 µM.

Based on the promising in vitro results, we next tested the most potent novel mitoK_Ca_3.1 inhibitor in vivo: we established a treatment scheme that did not affect the body weight of mice or induce visible organ damage (Figs. 5a, b, h and S5a). The daily administration of 3 nmol/g body weight (gbw) mitoTRAM-34 began 5 days after the subcutaneous injection of B16F10 cells into the flank of syngeneic C57BL/6J mice. The animals were sacrificed after 10 days of treatment (Fig. 5a). Tumor size in this model was reduced by ≈60% compared to vehicle-treated control mice (Fig. 5c). Because mitoTRAM-34 was also able to effectively kill PDAC cells and because the functional presence of mitoK_Ca_3.1 has previously been reported in a subset of pancreatic carcinoma cell lines [18], we corroborated the in vivo efficacy of our novel drug in a pancreatic cancer model as well. We orthotopically injected PAN-02 cells in the pancreas of syngeneic C57BL/6J mice, thus causing the development of pancreatic ductal adenocarcinomas [73], and used the same treatment scheme as above (Fig. 5a). Importantly, at sacrifice tumor volume and weight were significantly lower in mitoTRAM-34-treated animals than in vehicle-treated mice, underscoring the efficacy of this drug in multiple cancer models (Fig. 5d, e). Finally, we wondered whether the new TRAM-34 derivative would be able to decrease metastatic spread of melanoma cells in vivo, as expected from the in vitro results outlined in Fig. 4. For this purpose, B16F10 cells were injected into the footpad of mice and popliteal lymph node invasion [74] was analyzed after treatment with mitoTRAM-34. In two out of three vehicle-treated mice, black spots (indicating the presence of B16F10 melanoma cells) were clearly visible on the isolated lymph nodes, in contrast to lymph nodes from four mitoTRAM-34-treated mice (Fig. 5f). To obtain a more quantitative evaluation, cryosections of lymph nodes were stained with Melan-A, a melanocytic marker [75] (Fig. 5g). Importantly, at the applied dosage, the drug did not induce visible side effects or altered organ histology (Figs. 5h and S5a).

## Discussion

The K_Ca_3.1 channel has recently emerged as important target in the context of several diseases linked to altered apoptosis and migratory ability. Here we show that specific pharmacological targeting of the mitochondrial K_Ca_3.1 using the newly designed and synthesized mitoTRAM-34 in the µM range alters mitochondrial function and triggers cell death, while decreasing migration at sub-lethal doses. In vivo, mitoTRAM-34 was able to substantially reduce tumor volume of both melanoma and pancreatic ductal adenocarcinoma as well as melanoma metastasis. Importantly, no signs of toxicity were observed with the administration protocol we adopted.

In contrast to mitoTRAM-34, TRAM-34 had no or modest effects on various cancers in vivo (e.g., [76]). A K_Ca_3.1-mediated stress response was shown to be required for the survival of irradiated glioblastoma cells and a high transcript level was associated with a shorter overall survival time [77, 78] and a higher metastatic risk [79]. TRAM-34 was shown to reduce glioblastoma volume by 60% only when administered at high concentrations (120 mg/kg) for ten days [80]). In comparison, the dosage of mitoTRAM-34 used in our experiments was 3 nmol/gbw (corresponding to 2 mg/kg). Thus, mitochondria-targeting greatly enhances the efficacy of TRAM-34 both in vitro and in vivo, even though, as expected from studies of the affinity of TRAM-34 derivatives for K_Ca_3.1 [27], mitoTRAM-34 and TRAM-OH were less efficient blockers of the channel than TRAM-34 itself (Fig. 1d).

Mitochondria-targeted derivatives of TRAM-34 allow us to distinguish the pathophysiological roles of mitoK_Ca_3.1 in cancer cells from those of the plasma membrane-located channel [81], since mitochondriotropic drugs reach the organelle within a few minutes [82]. We found that in contrast to the membrane-impermeant K_Ca_3.1 inhibitor Maurotoxin [50], mitoTRAM-34 directly affected mitochondrial parameters such as membrane potential, ROS production, respiration, and morphology. These findings match well with what is known about different mitochondrial potassium channels, whose inhibition contributes to ROS production, volume regulation and to changes in membrane potential and in the rate of oxidative phosphorylation [1, 83, 84]. Blocking the entry of depolarizing cations such as K^+^ into the matrix is associated with enhanced ROS production which may lead to opening of the permeability transition pore, depolarization, matrix swelling, and cell death even independently of Bax/Bcl-2 function [85, 86]. Our data are in agreement with the study of Kovalenko and colleagues who found K_Ca_3.1 in the mitochondria of PDAC lines and reported that a decreased expression of K_Ca_3.1 as well as application of 10 µM rac-16, a membrane-permeant K_Ca_3.1 inhibitor, diminished oxidative phosphorylation and ATP production in several PDAC lines, suggesting a role of the channel in fine-tuning mitochondrial energy metabolism [18]. In accordance with their study and as expected, we found decreased respiration in the glycolytic B16F10 cells. Recently, a few studies pointed out that decyl-TPP^+^ (with no conjugation to an active molecule) can affect mitochondrial physiology [87]: by inserting into the IMM, decyl-TPP^+^ destabilizes complex I with a resulting increase in ROS generation and a reduced expression of the complex. Decyl-TPP^+^ also reduces membrane potential, OCR and ATP production [88] and enhances mitochondrial fragmentation as well as proton leak [87]. To counteract these effects, glycolytic ATP production is enhanced in order to avoid an energy crisis [88]. Although it has been clearly demonstrated that propyl-TPP^+^, the moiety used to generate mitoTRAM-34 here, does not produce any of these effects [87], and in our control experiments employing TPP-propyl-NH_3_ no such mitochondrial alterations have been observed, we cannot fully exclude a partial K_Ca_3.1-independent contribution of mitoTRAM-34 and *rev*-mitoTRAM to the observed effects, at least at the highest concentrations used.

Drastic changes in mitochondrial function, ultrastructure (with the loss of cristae), chromatin condensation and Annexin V binding upon mitoTRAM-34 treatment are compatible with apoptotic death of the cells. While at higher concentrations the drugs induce cell death, at sub-lethal sub-µM concentrations mitoTRAM-34 remarkably reduced cancer cell migration and colony formation. As mentioned above, K_Ca_3.1 has been linked to migration in several types of cancer [30] but whether mitoK_Ca_3.1 contributes to this process is still unknown. Here we show that the TRAM-34 derivatives decrease migration by downregulating BNIP-3 when applied at sub-lethal, sub-µM concentrations that causes mitochondrial fragmentation (Fig. 2f). BNIP-3 is a critical regulator of cell death whose enhanced expression has been linked to increased cell survival in several cell types. Maes and colleagues showed that in B16F10 melanoma cells BNIP-3 determines aggressive behavior and migration: downregulation of BNIP-3 prevented clonogenic growth, caused an increase of cellular ROS levels and led to alteration of the mitochondrial network [67]. Reduced BNIP-3 expression was accompanied by a reduction of the level of the cytoskeleton effector CDC-42 level, thereby linking BNIP-3 to cytoskeletal reorganization and migration. Our data obtained using mitochondriotropic K_Ca_3.1 inhibitors are in good agreement with these results. As mentioned above, one of the events that trigger a decrease of BNIP-3 expression is the activation of transcription factor NF-κB [69]. NF-κB subunit p65 can bind to the promoter of BNIP-3, repressing BNIP-3 transcription [89, 90]. We observed activation of redox-sensitive NF-κB by both our drugs. NF-κB can be activated by even a modest elevation of mitochondria-derived ROS [70, 91] that in the present study are released upon targeting mitoK_Ca_3.1 (Fig. 2c). Thus, a novel signaling pathway consisting in mitoK_Ca_3.1 inhibition/enhanced mitochondrial ROS production/NF-κB activation/BNIP-3 downregulation/CDC-42 inactivation/cytoskeleton reorganization/reduced migration can be proposed based on our data. The relevance of this pathway mediating the effects of mitoTRAM-34 on cell migration has also been confirmed using cells in which the activities/expression of these signaling molecules were modified either pharmacologically or genetically. When NF-κB activation or BNIP-3 downregulation or CDC-42 inactivation were prevented, the migration-reducing effect of mitoTRAM-34 was almost completely abolished. A possible role for HIF-1α [92] and AMPK, known to be linked to migration [93], downstream of our drugs has been excluded. Interestingly, K_Ca_3.1 activity has recently been linked to mitochondrial dysfunction through modulation of mitochondrial quality control via BNIP-3-mediated mitophagy [65]: K_Ca_3.1 deficiency rescued abnormal mitophagy by inhibiting BNIP-3 expression in diabetic mice. The exact link between channel function and BNIP-3 expression is, however, unknown.

Altogether, our study highlights an unexpected role for mitoK_Ca_3.1 in determining survival as well as migration and metastatic abilities of PDAC and melanoma cells. It remains to be determined whether the observed reduction of metastatic spread correlates mainly with primary tumor size reduction. Although the drugs were effective in triggering death and reducing migration of cancer cells in vitro and were efficient even in vivo against melanoma and PDAC, further work is required to improve their therapeutic index. Finally, it might be worthwhile to explore the effect of TRAM-34 derivatives on the tumor microenvironment, since K_Ca_3.1 function has been linked to the motility of T lymphocytes [94] and to the function of other immune cells [95].

## Supplementary information

Supplementary Figure Legends

Supplementary Figures

Original Data File

## Data Availability

All data generated or analyzed during this study are included in this published article and its supplementary information files.
