# Supplementary material for: Pharmacological targeting of the mitochondrial calcium-dependent potassium channel KCa3.1 triggers cell death and reduces tumor growth and metastasis in vivo
Source: Cell Death Dis. 2022 Dec 20;13(12):1055. doi: 10.1038/s41419-022-05463-8 (PMC9768205; doi:10.1038/s41419-022-05463-8)
Supplement: Supplementary file 1 — Supplementary Figure Legends [file 41419_2022_5463_MOESM1_ESM.docx]

**SUPPLEMENTARY FIGURE LEGENDS, Bachmann et al.**

**Fig. S1. Docking poses of TRAM-34 and derivatives in KcsA- and Kv1.2-based K_Ca_3.1 models. a)** Docking pose for mitoTRAM-34, TRAM-34-OH and TRAM-34 in the KcsA-based K_Ca_3.1 model. The corresponding docking scores are shown in Fig. S1b. **b)** Best docking poses found for mitoTRAM-34, TRAM-34-OH and TRAM-34 in the Kv1.2-based K_Ca_3.1 model. The docking scores are given in the table inset. **c)** Superimposition of the best docking poses found for three different molecules in two models of K_Ca_3.1 (Kv1.2-based on the left and KcsA- based on the right). The corresponding docking scores are given in the table inset.

**Fig. S2. Effects of mitoTRAM-34 and *rev*-mitoTRAM on mitochondrial physiology. a)** Images of mitochondrial membrane potential changes (left) and superoxide production (right) in B16F10 cells visualized by changes in TMRM or mitoSOX fluorescence, respectively, upon addition of TRAM-34-OH (T.-OH), the triphenylphosphonium group (TPPP-NH_3_) or membrane-impermeant K_Ca_3.1 inhibitor maurotoxin at the indicated concentrations. The quantification of the fluorescence signal is shown below. Fluorescence is expressed as percentage of the initial intensity (mean + SEM; ordinary Two-Way Anova with Dunnett’s posttest. N=3. * p < 0.05, ** p < 0.01 compared to control). **b)** Effect of mitoTRAM-34 and *rev*-mitoTRAM on the Oxygen Consumption Rate (OCR) of B16F10 cells. Values were normalized with respect to basal respiration recorded before compound addition. Arrows indicate the addition of the tested compound, oligomycin (oligo), FCCP and antimycin A (anti). Mean + SEM from N=3-4 are shown. **c)** Scatter plots showing mitochondrial bioenergetic parameters calculated from the OCR shown in b) (mean + SEM, N=3-4. Ordinary One-Way Anova with Dunnett’s posttest. N=3-4. ** p < 0.01 compared to control). **d-e)** Activity of complex V in isolated B16F10 (d) or mouse liver (e) mitochondria after treatment with different concentrations of mitoTRAM-34 (mitoT.), *rev*-mitoTRAM (*rev*-m.), TRAM-34-OH, TRAM-34 or TPPP-NH_3_ as indicated in the graphs. The activity was calculated from the slope of NADH absorbance over time (mean + SEM. Kruskal- Wallis test with Dunn’s posttest. N=3-5). **f)** ATP synthesis over time in isolated mouse liver mitochondria incubated with the indicated compounds. Oligomycin was used as positive control and to subtract background luminescence. In presence of TRAM-34 derivatives, ATP synthesis does not change compared to control, indicating a specific effect on ATP hydrolysis rather than ATP synthesis. Mean + SEM of N=3 are shown. **g)** As a control for complex V activity experiments, pyruvate kinase (PK) and lactate dehydrogenase (LDH) activity was assayed in spectrophotometric experiments. NADH absorbance decreases after addition of ADP. The slope does not change in presence of different TRAM-34 derivatives, ruling out an effect of the latter on PK or LDH activity that may affect the results regarding complex V inhibition. **h)** Complex I activity in isolated mouse liver mitochondria incubated with different concentrations of mitoTRAM-34. Rotenone was used to subtract background absorbance. The activity was calculated from the slope of NADH absorbance over time (mean + SEM. No significant differences were detected. N=2). **i)** Complex III activity in isolated mouse liver mitochondria incubated with different concentrations of mitoTRAM-34. Antimycin A was used to subtract background absorbance. The activity was calculated from the slope of absorbance of oxidized cytochrome *c* over time (mean + SEM. No significant differences were detected. N=6).

**Fig. S3. The novel TRAM-34 derivatives dose-dependently induce cell death in a variety of cancer cells. a)** Dose-response curve showing the sensitivity of B16F10 cells to TRAM-34-OH, as assessed by MTS assays. Shown are mean + SEM and nonlinear fit curve of N=4. **b)** Lack of toxicity of TRAM-34 and TPPP-NH_3_ as assayed in MTS assays on B16F10 cells. Cells were treated for 24 h (mean + SEM. N=3). **c)** Representative images of an Annexin V-FITC cell death assay. B16F10 cells were treated for 24 h with TRAM-34, TRAM-34- OH (T.-OH), TPPP-NH_3_ or staurosporine (positive control) at the indicated concentrations and apoptotic cells were stained with Annexin V-FITC. Bright field images, FITC fluorescence, Hoechst staining and the merged images are shown. Scale bar is 25 µm. **d)** Quantification of Annexin V-FITC staining shown in c). The number of Annexin V-positive cells on total cell count is shown (mean + SEM, ordinary One-Way Anova with Dunnett’s posttest. N=3. ** p < 0.01). **e)** Dose-dependency of mitochondrial superoxide production and loss of membrane potential as indicated by the fluorescence intensity of mitoSOX and TMRM, respectively, in B16F10 cells upon addition of *rev*-mitoTRAM at different concentrations. Fluorescence is expressed as percentage of the initial intensity (mean + SEM; ordinary Two-Way Anova, N=2, p-value is indicated). **f)** Dose- response curves showing the sensitivity of B16F10 cells to mitoTRAM-34 and *rev*-mitoTRAM in normoxia vs hypoxia. No difference was detected upon mitoTRAM-34 treatment, while *rev*-mitoTRAM was slightly less effective in hypoxic conditions (mean + SEM with nonlinear fit, N=3-4. Ordinary Two-Way Anova. p-value is indicated).

**Fig. S4. Sublethal doses of mitoTRAM-34 and rev-mitoTRAM reduce migration and anoikis resistance of B16F10 cells by affecting the cytoskeletal architecture. a)** Representative images of wound healing assays also shown in Fig. 4a at increased magnification in B16F10 cells. Cells were treated with 0.5 µM mitoTRAM- 34 (mitoT.), 5 µM rev-mitoTRAM (rev-m.) or 10 µM TRAM-34. The area of the gap was highlighted in cyan for better visualization. Scale bar is 100 µm. **b)** The amount of ATP compared to the control in B16F10 cells treated for 2 h as indicated. 1 µg/ml oligomycin was used as positive control. Cells were cultured either in glucose or in galactose (that pushes cells towards oxidative phosphorylation) to distinguish between total cellular and mitochondrial ATP production. Mean + SEM of N=4 are shown (One-sample T test. * p < 0.05). **c)** Quantification of the Western Blot shown in Fig. 4c. β-actin was used for normalization and protein expression in treated samples is expressed as % compared to control (mean + SEM, N=4. One-Sample T test. ns = not significant). **d)** Quantification of the Western Blots shown in Fig. 4d. β-actin was used for normalization and protein expression in treated samples is expressed as % compared to control (mean + SEM, N=4. One-Sample T test. ns = not significant). **e)** Expression of genes related to mitochondrial transcription in B16F10 cells after treatment for 24 h as indicated, analysed by qRT-PCR. The normalized expression level relative to the control is reported (mean + SEM, N=3. Ordinary One-Way Anova with Dunnett’s posttest. ns: not significant). **f)** Quantification of the Western Blot shown in Fig. 4f. β-actin was used for normalization and protein expression in treated samples is expressed as % compared to control (mean + SEM, N=4. One-Sample T test. ns = not significant). **g)** Additional confocal images of phalloidin staining in B16F10 cells treated for 24 h with 0.5 µM mitoTRAM-34 (mitoT.) or 5 µM rev-mitoTRAM (rev-m.). Multiple filopodial extensions are visible in control cells (indicated by white arrows) that are mostly lacking in treated samples. Scale bar is 15 µm. **h)** Representative images of wound healing assays in B16F10 cells quantified in Fig. 4m. **i)** Representative Western Blots showing overexpressed levels of BNIP-3 in B16F10 (upper panel) and MDA-MB-231 (lower panel) cells at the timepoints analysed in wound healing assays (15-32 hours after performing the scratch). Vinculin was used as loading control. **j)** Quantification of wound scratch assays in MDA-MB-231 cells. Cells were treated with 0.5 µM mitoTRAM-34 (mitoT.) with or without BNIP-3 overexpression (OE) or the Rho/Rac/Cdc42 Activator I (1 μg/ml). The gap area was measured 0, 15, 24 and 32 h after performing the scratch using ImageJ and expressed as % of the initial gap area (mean + SEM, Two-Way Anova with Dunn’s posttest. N=3. P-value compared to control is indicated).

**Fig. S5. The novel TRAM-34 derivates reduce tumor growth in vivo in a melanoma and pancreatic cancer model. a)** Representative images of different organs from mice treated with the vehicle alone or mitoTRAM- 34, stained with H&E. Scale bar is 500 µm.
