# Supplementary figures and images for: Pharmacological targeting of the mitochondrial calcium-dependent potassium channel KCa3.1 triggers cell death and reduces tumor growth and metastasis in vivo
Source: Cell Death Dis. 2022 Dec 20;13(12):1055. doi: 10.1038/s41419-022-05463-8 (PMC9768205; doi:10.1038/s41419-022-05463-8)

Fig. S1

a

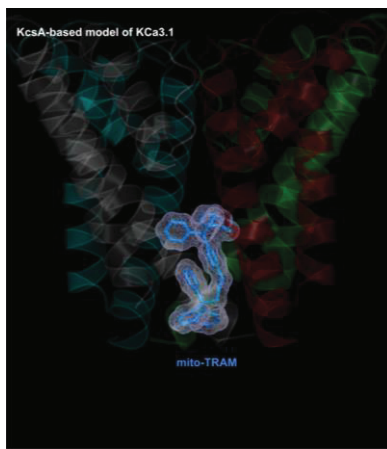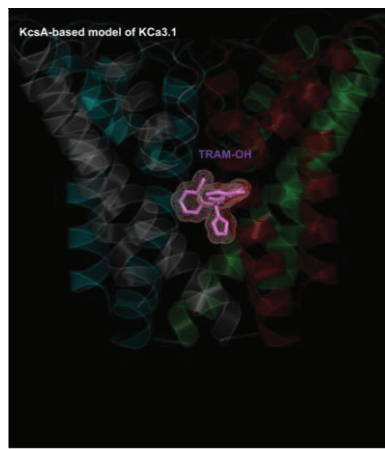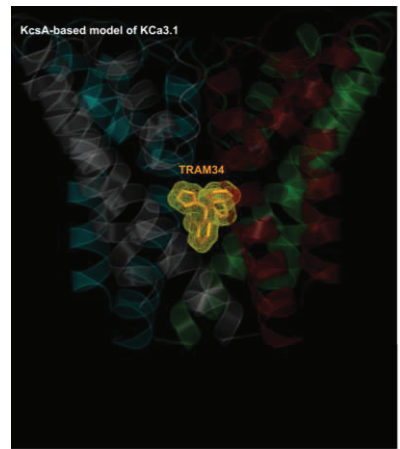

b

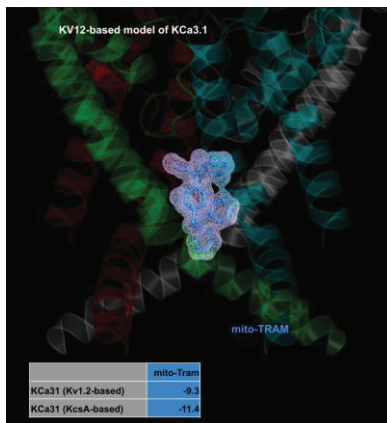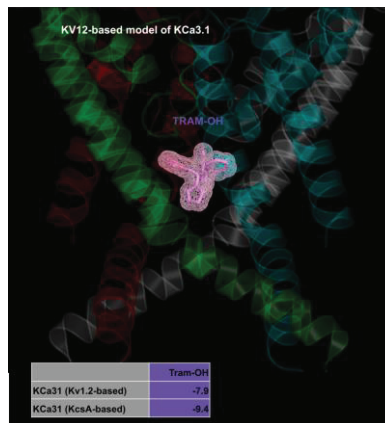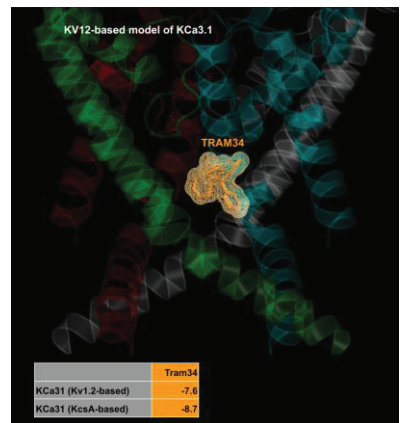

c

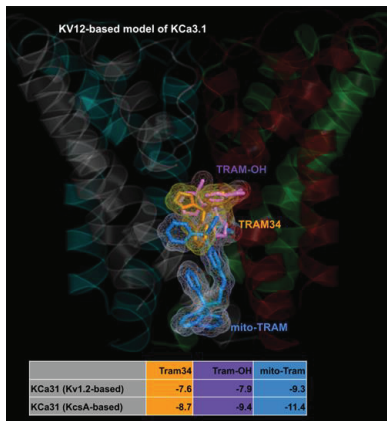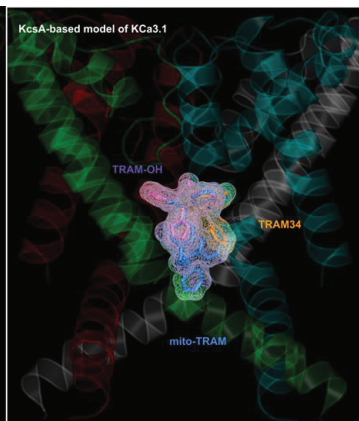

**Fig. S2**

**a**

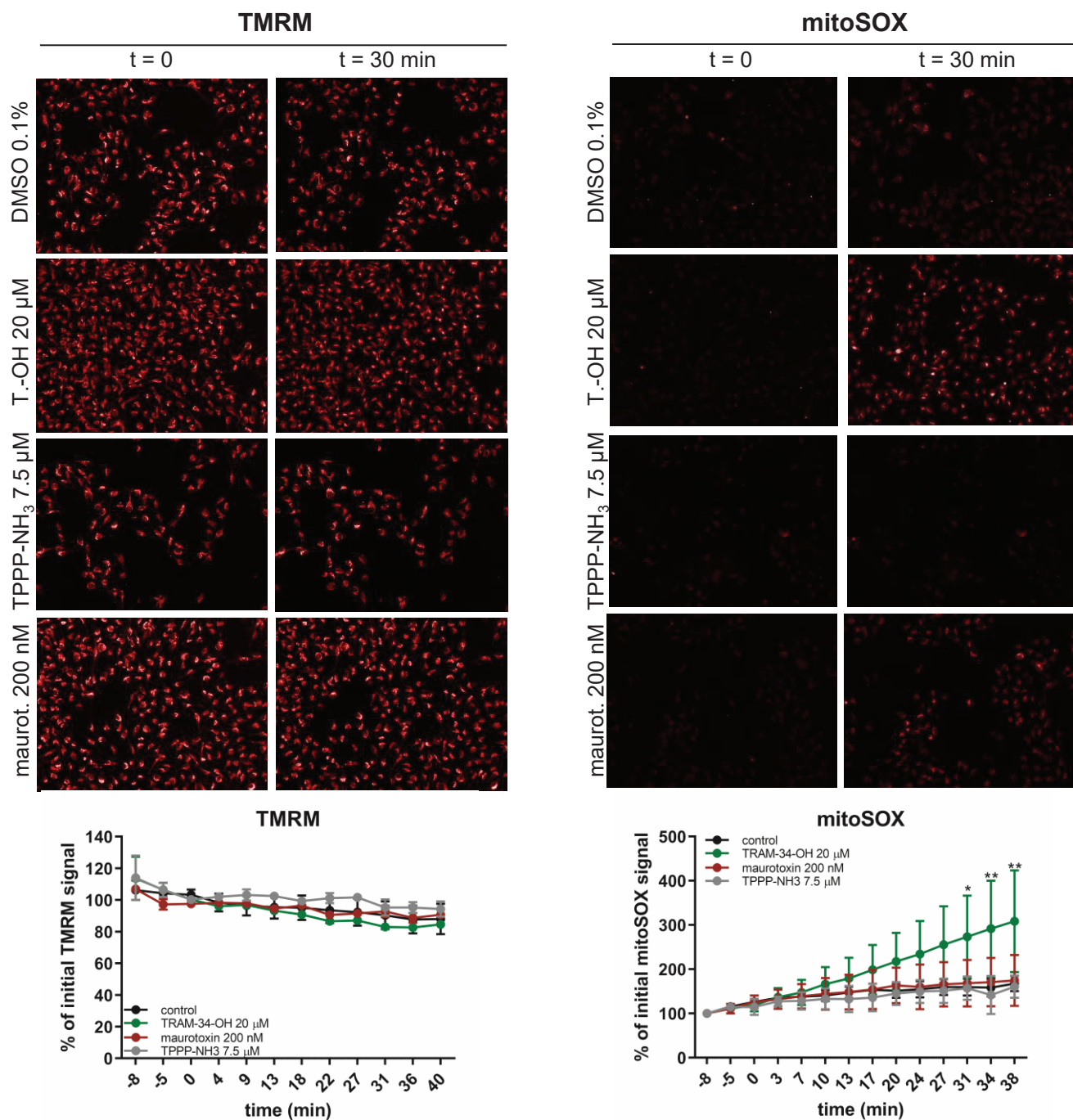

**b**

**c**

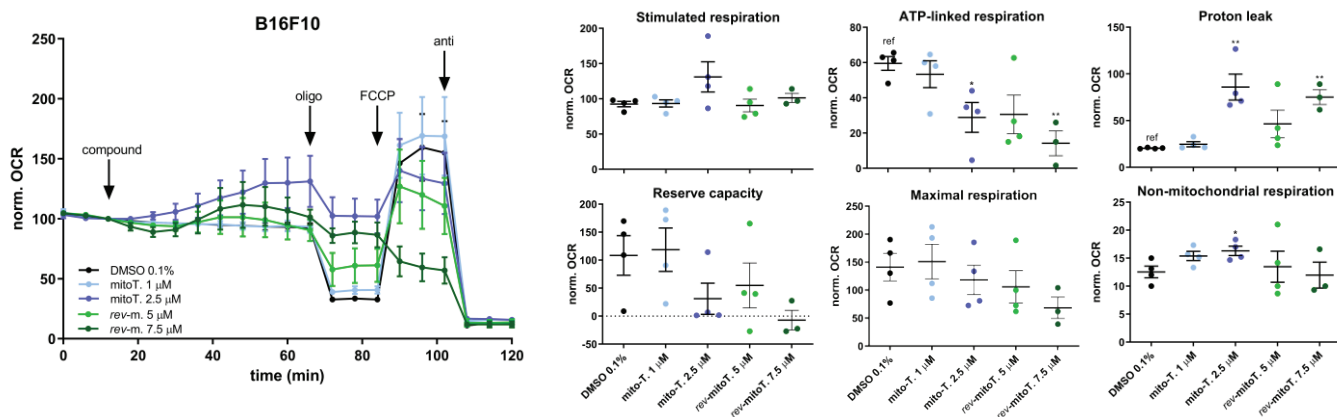

d

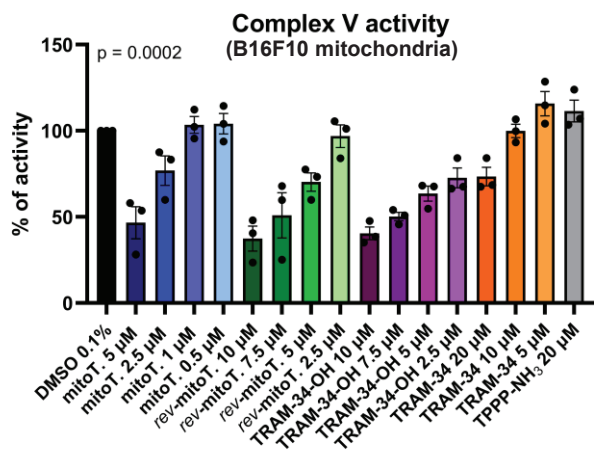

e

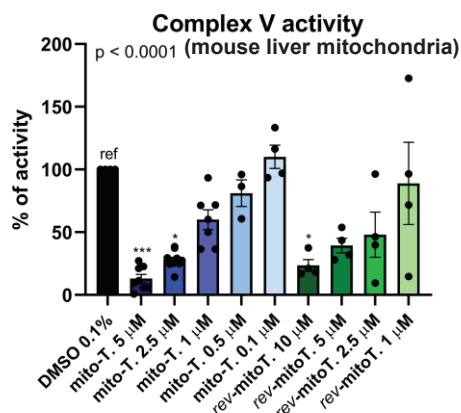

f

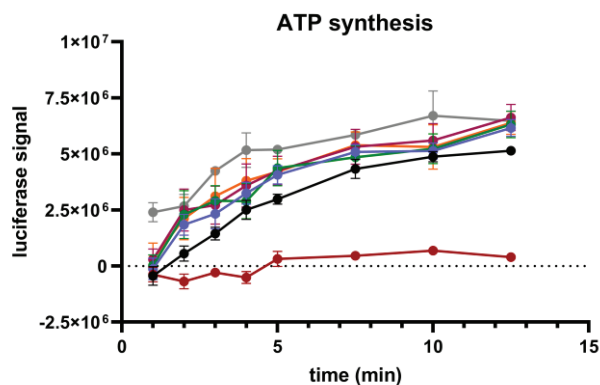

h

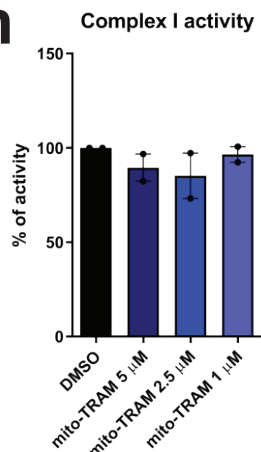

g

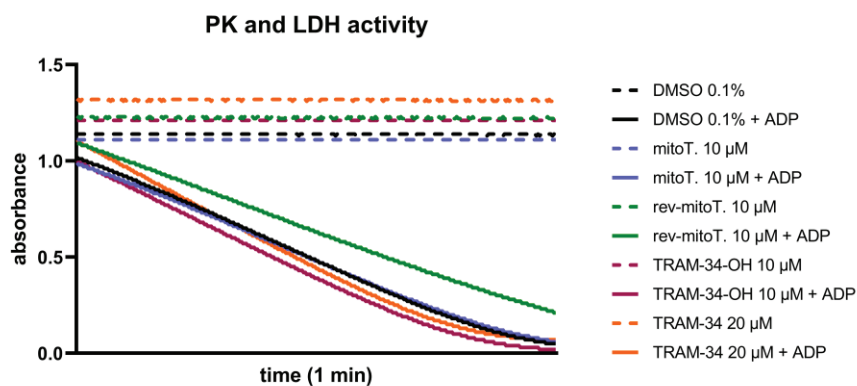

i

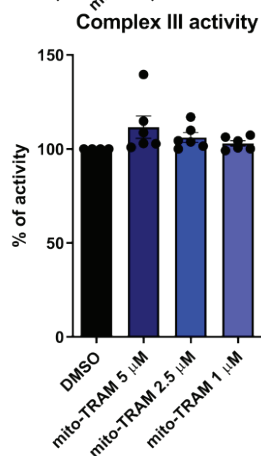

**Fig. S3****a**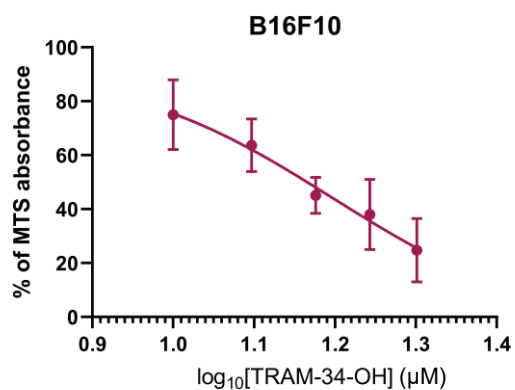**b**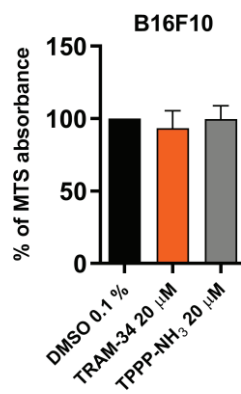**c**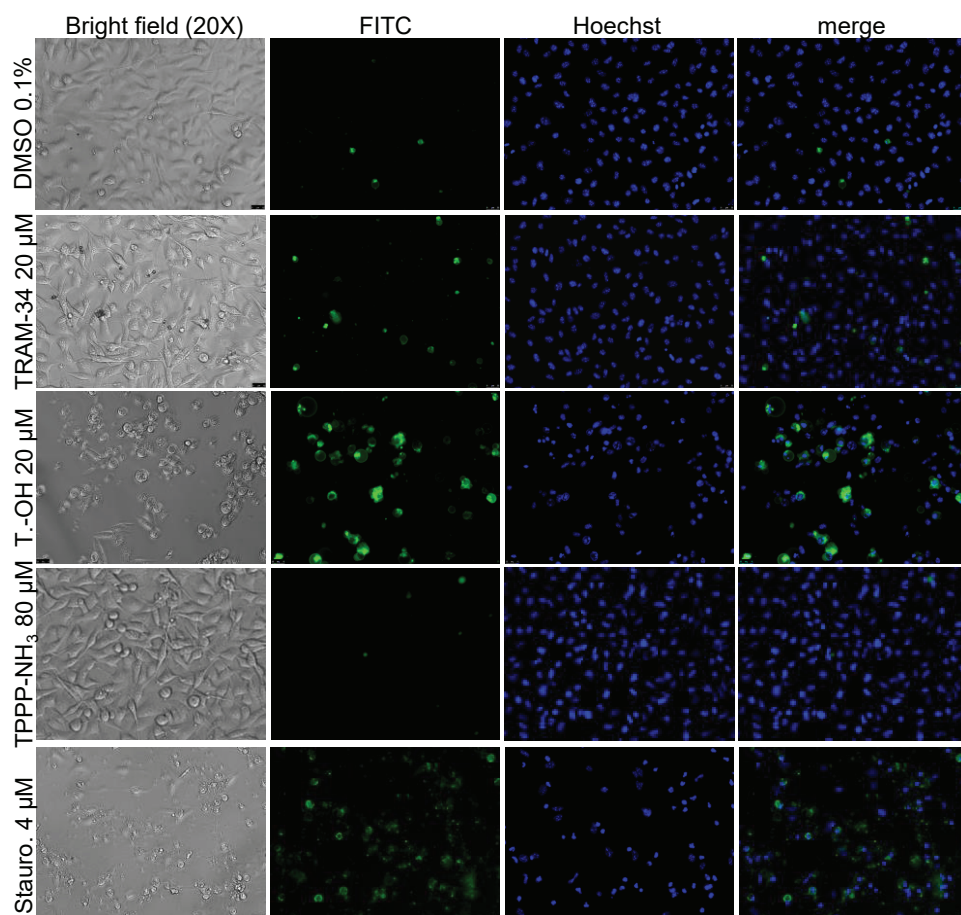**d**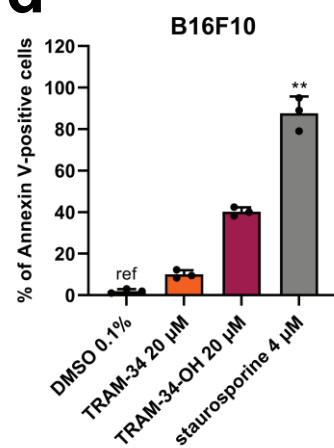**e**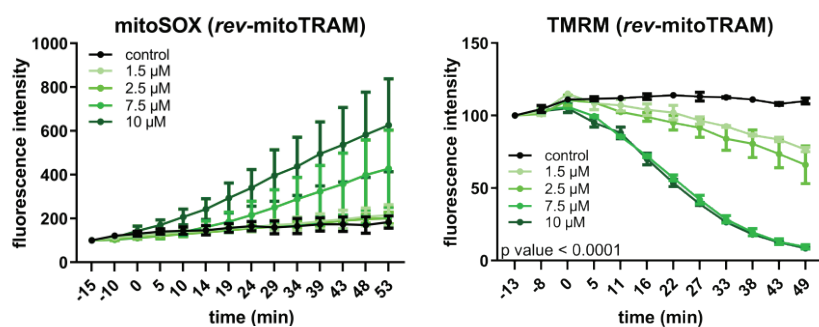**f**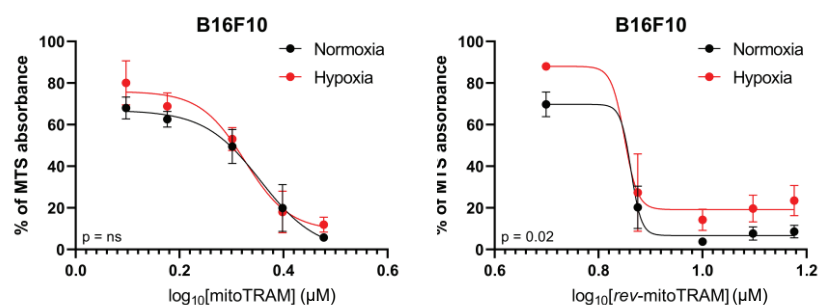

**Fig. S4**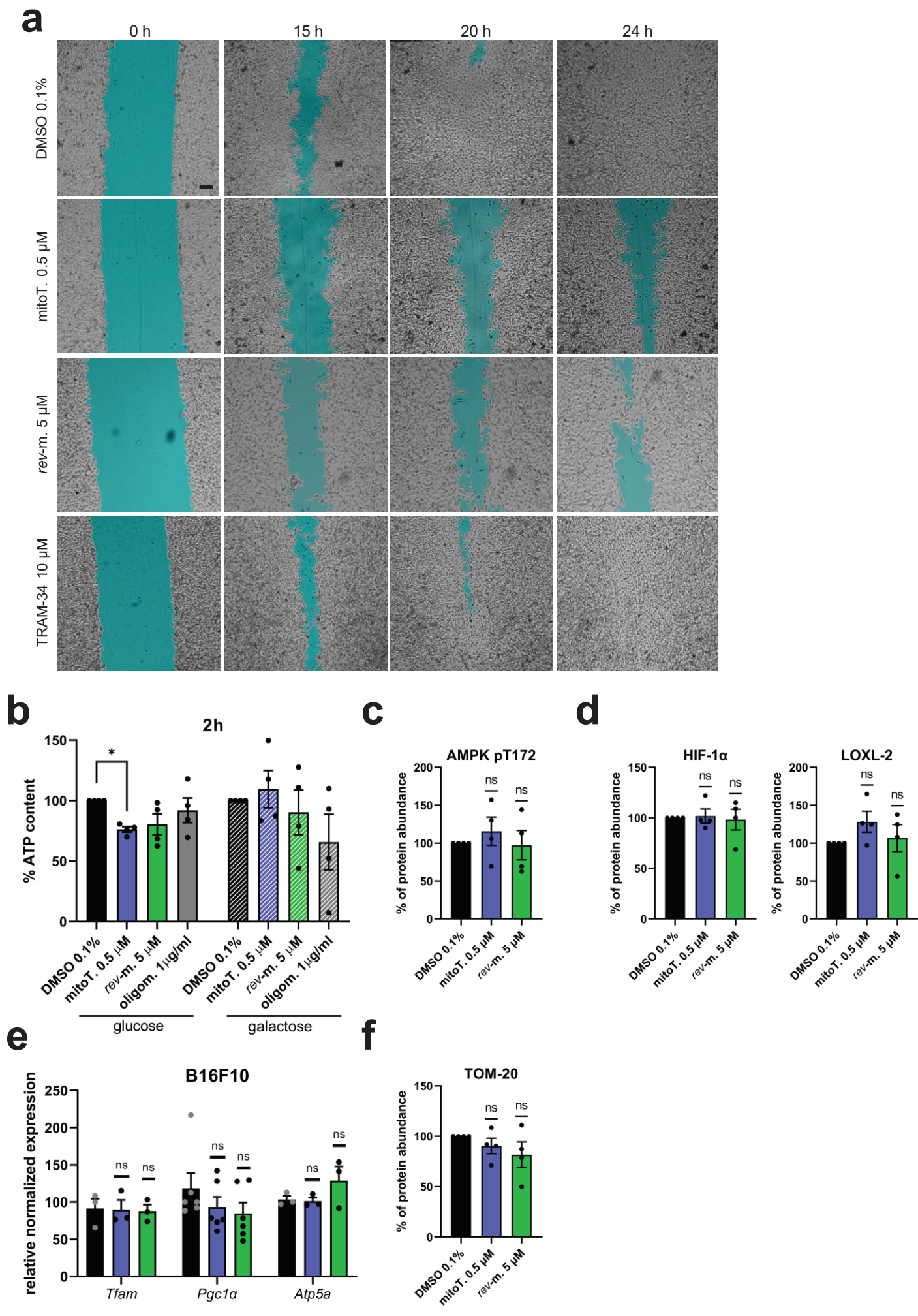

**Fig. S4****g**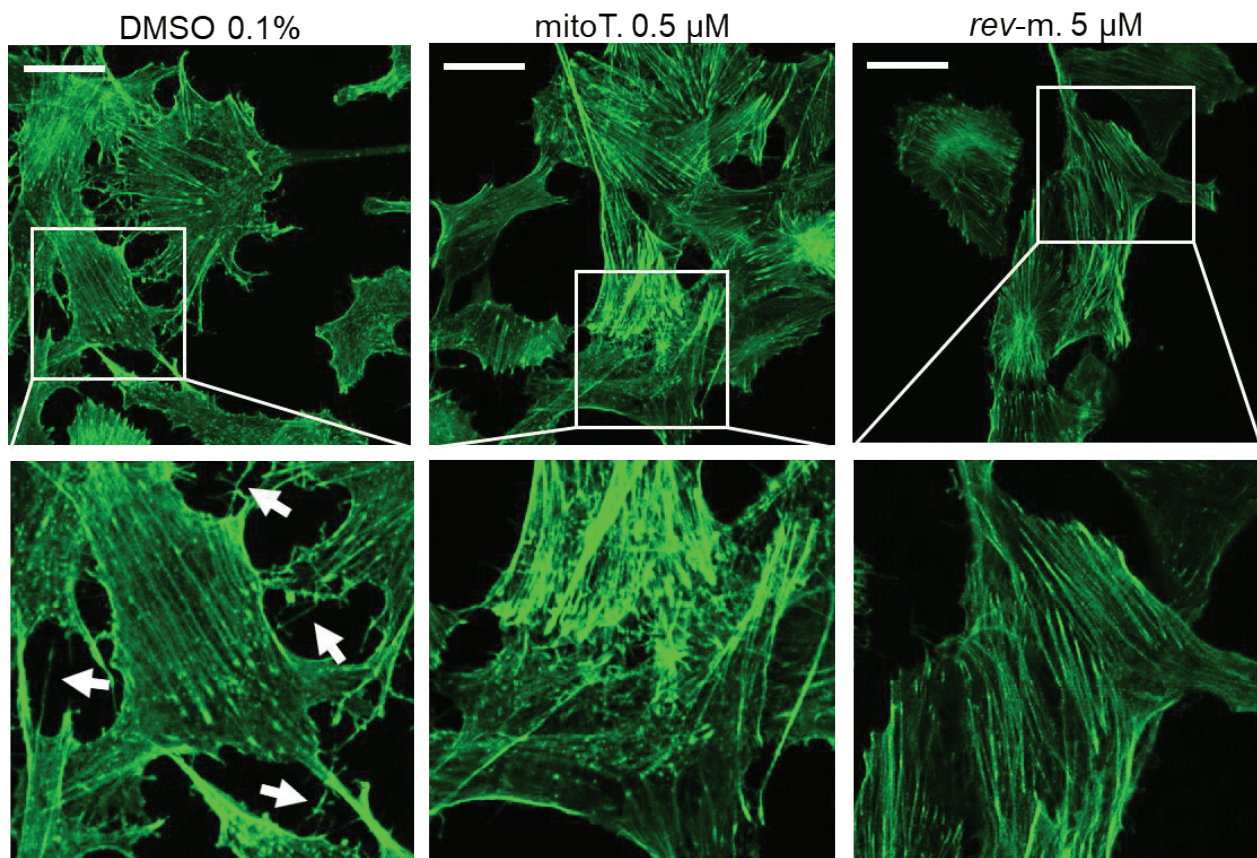**h**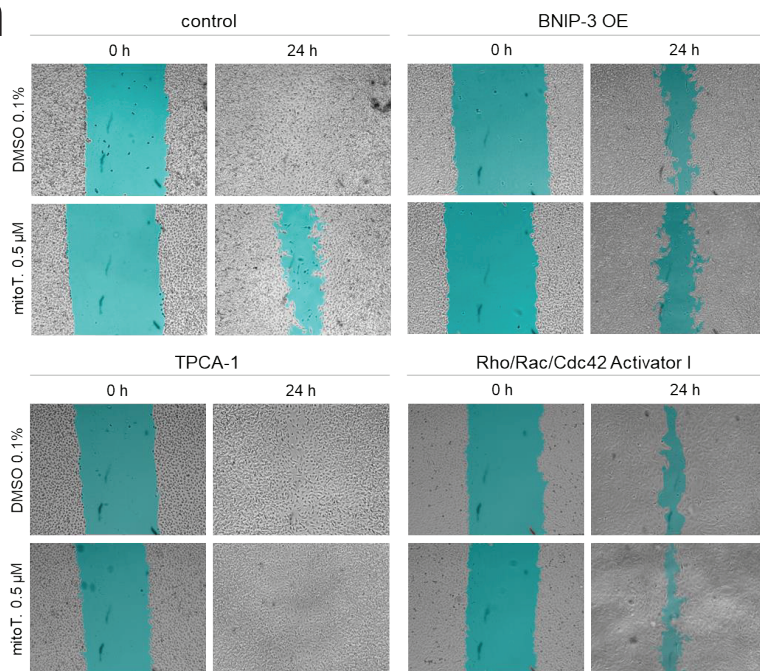**i**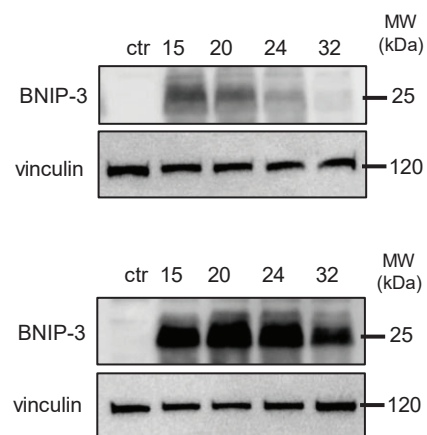**j**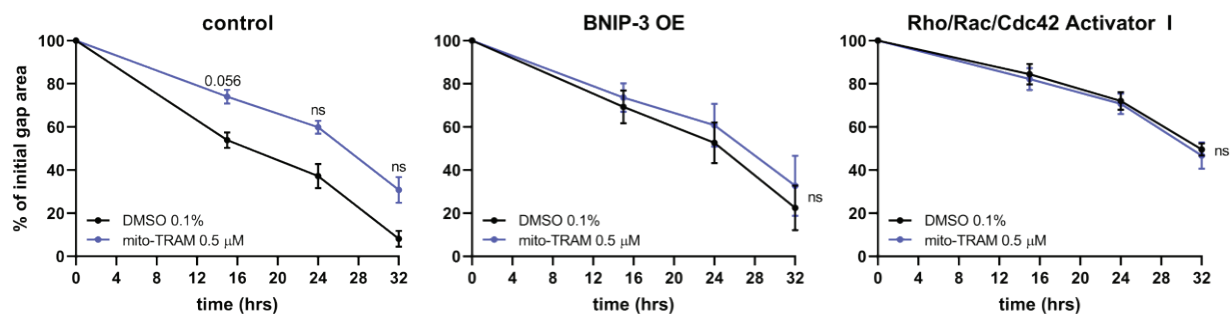

**Fig. S5**

**a**

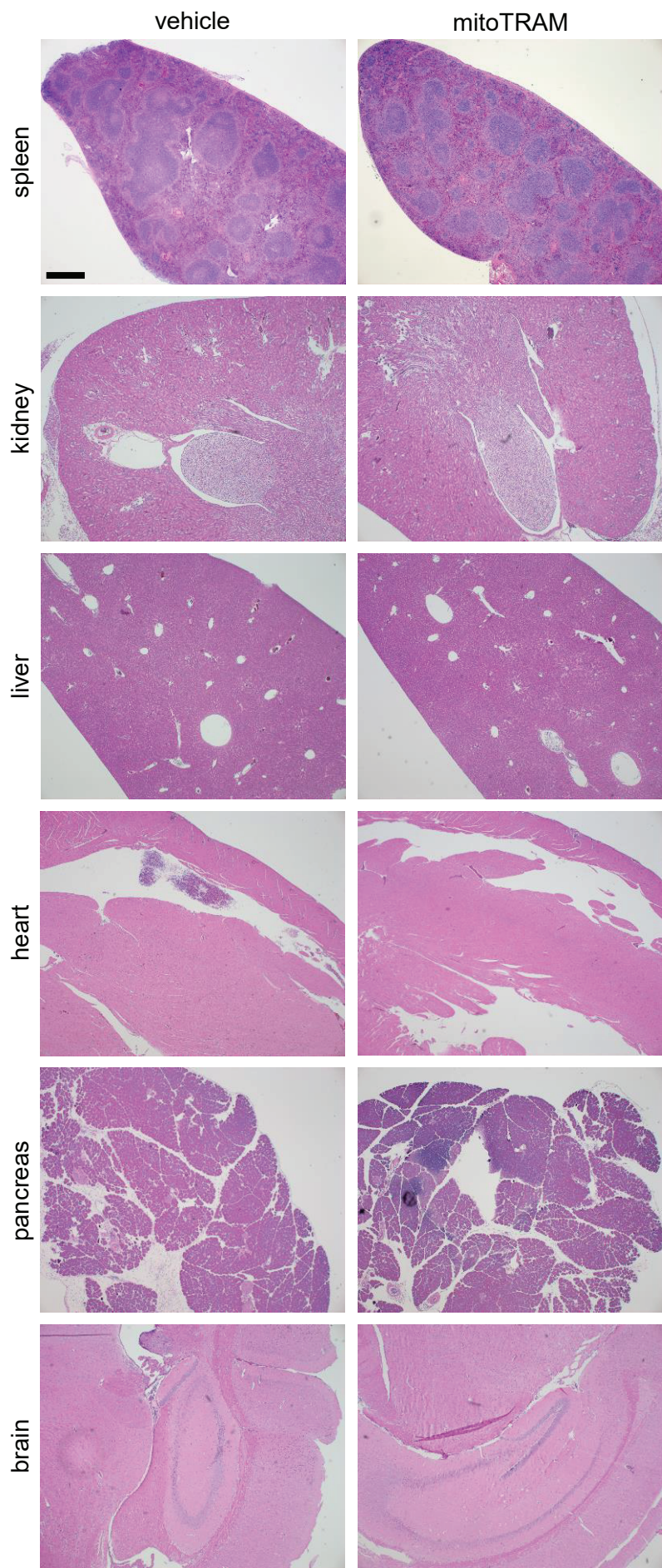

Supplement: Supplementary file 2 — Supplementary Figures [file 41419_2022_5463_MOESM2_ESM.pdf]
