# Supplementary material for: Pharmacological targeting of the mitochondrial calcium-dependent potassium channel KCa3.1 triggers cell death and reduces tumor growth and metastasis in vivo
Source: Cell Death Dis. 2022 Dec 20;13(12):1055. doi: 10.1038/s41419-022-05463-8 (PMC9768205; doi:10.1038/s41419-022-05463-8)
Supplement: Supplementary file 3 — Original Data File [file 41419_2022_5463_MOESM3_ESM.pdf]

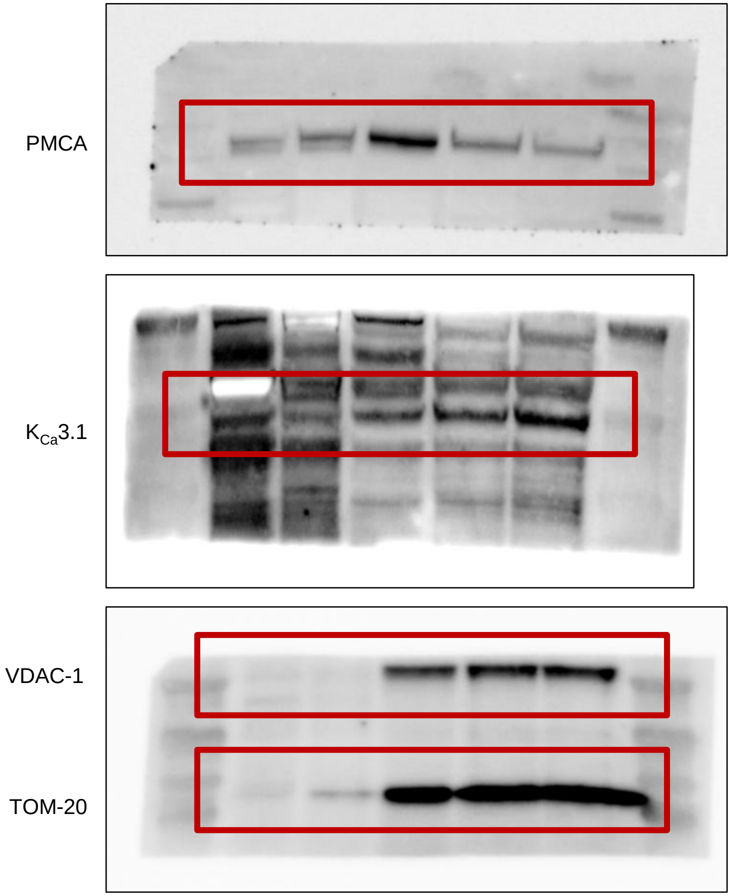
PMCA
$K_{Ca}3.1$
VDAC-1
TOM-20

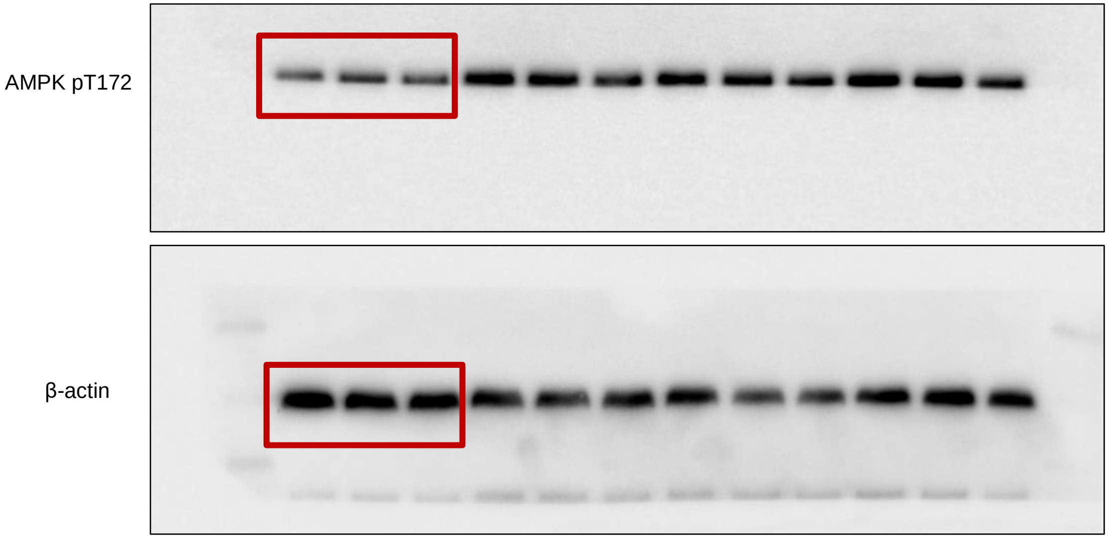
AMPK pT172
β-actin

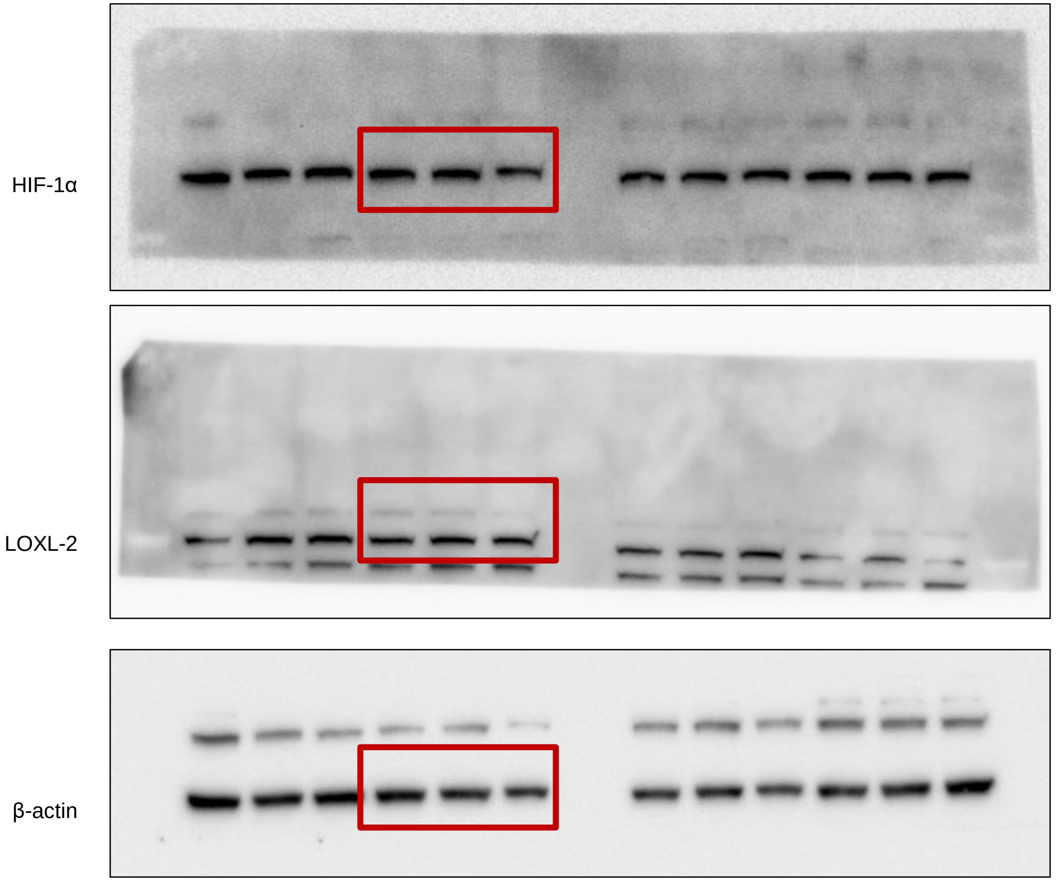
HIF-1α
LOXL-2
β-actin

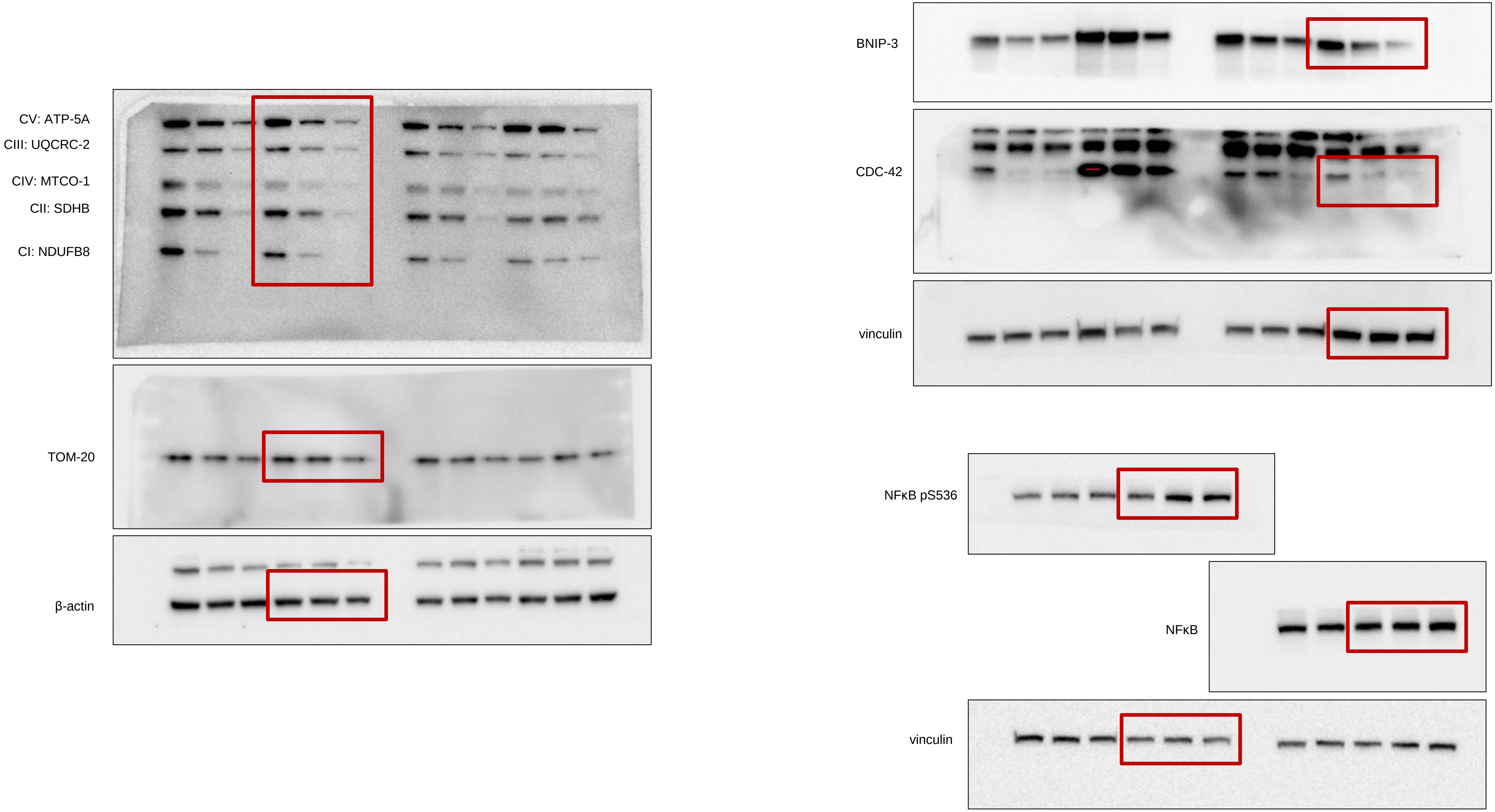
CV: ATP-5A
CIII: UQCRC-2
CIV: MTCO-1
CII: SDHB
CI: NDUFB8
TOM-20
β-actin
BNIP-3
CDC-42
vinculin
NFκB pS536
NFκB
vinculin

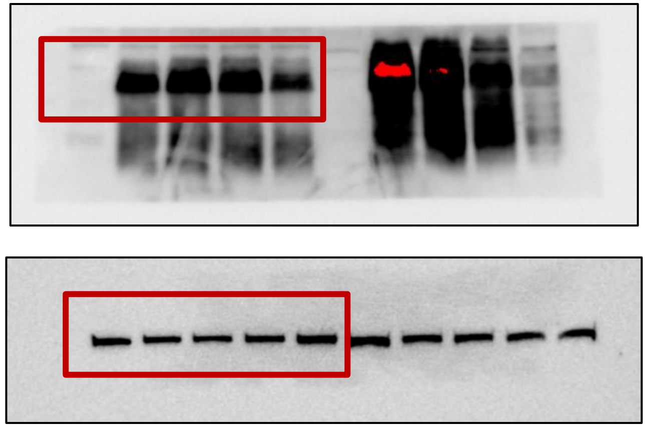

BNIP-3

vinculin

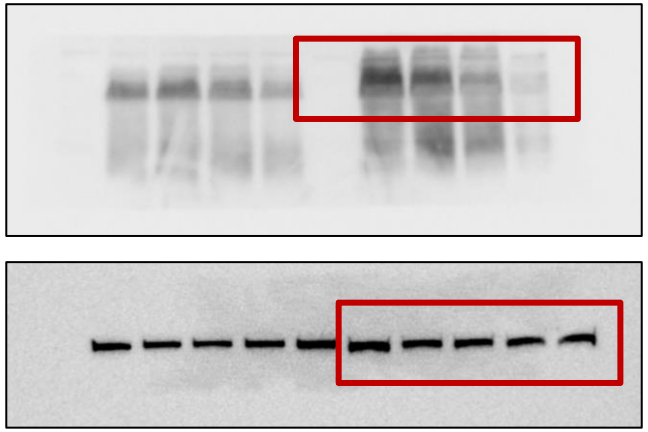

BNIP-3

vinculin
